# Cryo-EM structure of the QseG-QseE complex reveals an accessory protein-driven two-component system activation mechanism

**DOI:** 10.1128/mbio.02864-25

**Published:** 2025-11-17

**Authors:** Piqian Gong, Guobang Li, Weixun Li, Mengyuan Xu, Xuyao Jiao, Xudong Chen, Beile Gao, Xiang Gao

**Affiliations:** 1 State Key Laboratory of Microbial Technology, Shandong University 214177 , Qingdao, China; 2 Institutes of Biomedical Sciences, College of Life Sciences, Inner Mongolia University 12576 , Hohhot, China; 3 Ministry of Education Key Laboratory of Protein Science, Tsinghua-Peking Center for Life Sciences, Beijing Advanced Innovation Center for Structural Biology, School of Life Sciences, Tsinghua University 98441, Beijing, China; 4 State Key Laboratory of Tropical Oceanography, Guangdong Provincial Key Laboratory of Applied Marine Biology, South China Sea Institute of Oceanology, Chinese Academy of Sciences 555069 , Guangzhou, China; UT Southwestern Medical Center, Dallas, Texas, USA

**Keywords:** two-component system, signal transduction, Cyro-EM structure, accessory protein, QseEGF

## Abstract

The two-component system (TCS) enables bacteria to sense and respond to environmental changes through histidine kinase-mediated signaling cascades. Although the core components of TCSs have been extensively studied, the molecular basis of accessory proteins in modulating histidine kinase activity remains poorly understood. Here, we report that the outer membrane lipoprotein QseG functions as an accessory protein that directly binds to and activates the histidine kinase QseE via its C-terminal domain. Cryo-electron microscopy (Cryo-EM) structural analysis of the QseG-QseE complex reveals a novel yet conserved interaction mode between an accessory lipoprotein and a histidine kinase, which bridges the outer membrane to cytoplasm. Furthermore, systematic truncation assays and photo-crosslinking experiments indicate that outer membrane-anchored QseG is sufficient and prone to engage with and activate the inner membrane histidine kinase QseE under cultured conditions. Our findings provide mechanistic details for accessory lipoprotein-mediated TCS activation, expanding our understanding of bacterial signaling. The evolutionary conservation of this interaction across bacterial pathogens underscores its broad biological significance and potential as a therapeutic target.

**IMPORTANCE:**

The classical TCS system in bacterial signal transduction is composed of two proteins—a histidine kinase and its cognate response regulator. More and more studies have revealed the presence of accessory proteins that can modulate the histidine kinase activity and affect signal transduction, but their mechanisms remain largely elusive. This study unveils a previously unrecognized mechanism by which bacterial accessory lipoproteins mediate TCS activation. We provide compelling evidence that QseG directly interacts with QseE through an evolutionarily conserved structural interface, readily and sufficiently activating QseE’s autokinase activity and downstream signaling. Given the essential role of QseEF in bacterial virulence and stress adaptation, our findings pave the way for the development of antimicrobial strategies targeting this conserved lipoprotein-mediated activation mechanism.

## INTRODUCTION

Two-component systems (TCSs) are essential for bacterial adaptation, regulating gene expression to modulate various physiological processes in response to environmental cues (1, 2). A canonical TCS consists of a transmembrane histidine kinase (HK) sensor and its cognate cytoplasmic response regulator (RR), functioning through a conserved phosphorelay mechanism (3, 4). The N-terminal sensory domains of many HKs are typically localized to the periplasm or transmembrane regions, where they detect physicochemical signals such as nutrient availability, osmotic pressure, and redox potential (5–8). In addition, their C-terminal kinase domains mediate signal transduction via three enzymatic activities: autokinase activation, phosphotransfer to RRs, and phosphatase-dependent signal termination (9, 10).

Beyond these two core components, accessory proteins play a pivotal role in refining signal perception, enabling the formation of three-component or multi-component regulatory networks (11–14). For a subset of inner membrane-localized HKs, accessory proteins modulate kinase activity by establishing spatially organized networks across the inner membrane, periplasm, and outer membrane through strict subcellular localization (11). Inner membrane-associated accessory proteins typically bind specific signaling molecules and interact directly with HKs to regulate kinase activity (15, 16). Periplasmic accessory proteins also primarily act as sensory proteins, binding extracellular ligands and transmitting signals to HKs, thereby enhancing environmental signal perception (17–20). In addition, few studies have reported outer membrane-associated accessory proteins that serve as initial sensors, detecting envelope stress signals and modulating TCS activity via interactions with HKs or other signaling proteins (21–23). Despite these insights, the structural basis of accessory protein-mediated HK activation remains largely unexplored.

QseEF, a TCS responsive to multiple ligands including phosphate, sulfate, adrenaline, and AI-3, is essential for the virulence of pathogenic *Enterobacteriaceae* (24–27). A recent study has shown that QseEF plays a crucial role in amino sugar biosynthesis by upregulating small RNA *glmY*, thereby maintaining cell membrane homeostasis (28). Notably, genomic analyses reveal that a third gene *qseG* is co-transcribed with *qseE* and *qseF* (29), encoding an outer membrane lipoprotein anchored to the inner leaflet via N-terminal lipid modification (27, 30). Previous studies have demonstrated the periplasmic interaction between QseG and QseE and its essential role in downstream signaling (28); however, the precise molecular mechanism by which QseG activates QseE remains unknown.

Here, we demonstrate that QseG functions as a concentration-dependent activator that enhances QseE autokinase activity through direct interaction. Cryo-EM analysis of the QseG-QseE complex, combined with bioinformatics, reveals a novel yet evolutionarily conserved accessory protein-kinase interaction mode that is widely distributed across *Pseudomonadota*. Systematic truncation assays combined with photo-crosslinking experiments further demonstrate that the outer membrane-anchored QseG efficiently binds to and activates QseE under standard culture conditions. Collectively, our findings establish QseG as an endogenous activator of the QseEF signaling pathway via a conserved regulatory mechanism. This unique activation strategy not only underscores the specialized role of QseG but also provides a potential target for antivirulence therapeutics aimed at disrupting this critical kinase-regulator interaction.

## RESULTS

### The *qseEGF* gene cluster is widespread across *Pseudomonadota*

**Fig 1:**
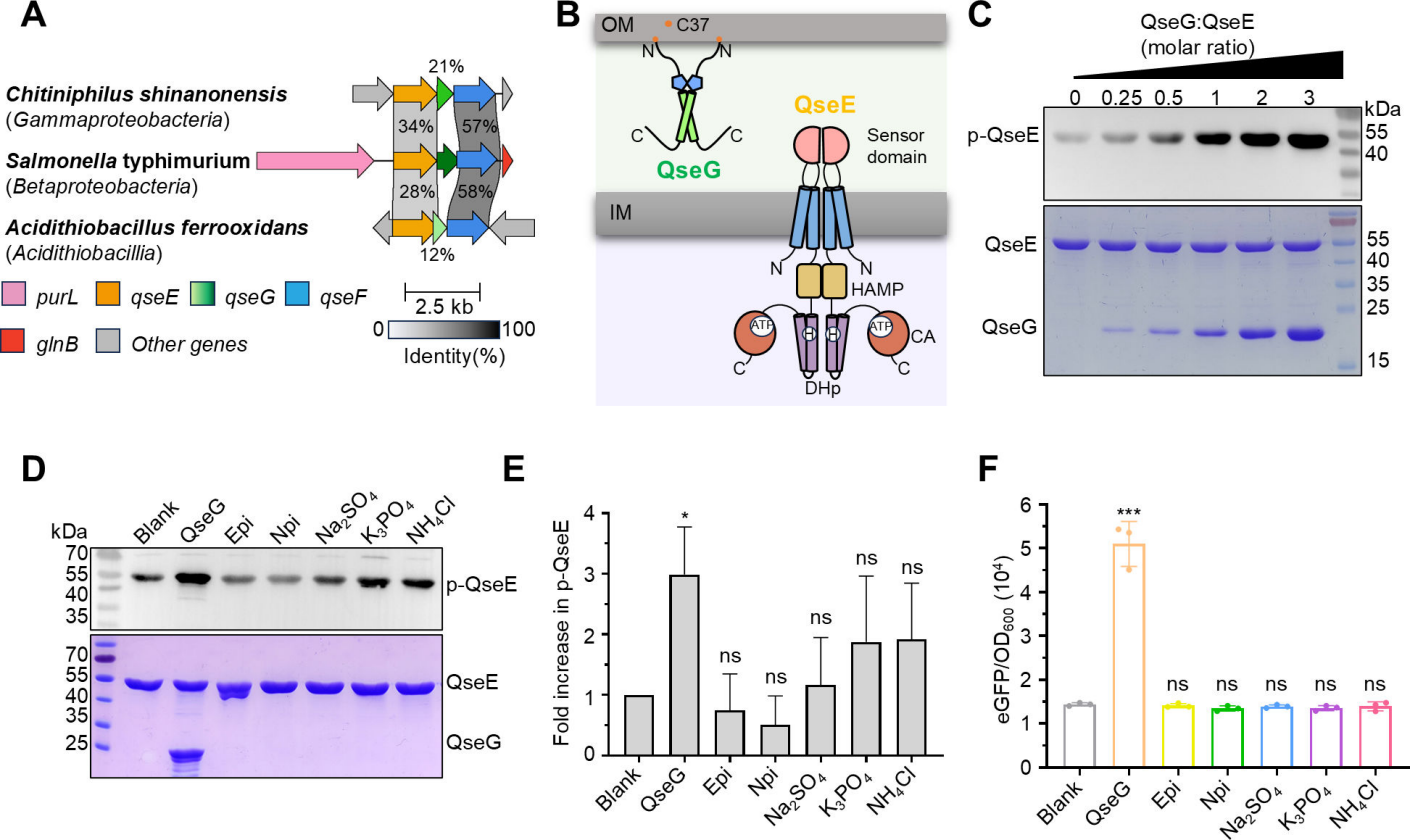
QseG acts as a direct activator of QseE. (**A**) Schematic representation of the *qseEGF* locus in three representative bacteria. The percent amino acid identities of full-length QseE, QseG, and QseF between *S.* Typhimurium, *Chitiniphilus shinanonensis*, and *Acidithiobacillus ferrooxidans* are indicated. ORFs with different predicted functions are color-coded: *purL* (pink), *qseE* (orange), *qseG* (gradient green), *qseF* (blue), *glnB* (red), and non-conserved genes (gray). (**B**) Diagram of QseG and QseE subcellular localization. QseG is a lipoprotein anchored to the inner leaflet of the outer membrane via N-terminal lipid modification, and QseE is a histidine kinase localized in the inner membrane. The orange dot designates the lipidated cysteine at position 37, following signal peptide cleavage. (**C**) Western blot analysis of QseE autokinase activity in response to varying QseG concentrations. The QseG and QseE proteins were mixed at the indicated molar ratios (spanning from 0:1 to 3:1) before adding the substrates ATP-γ-S. Catalyzed by the QseG-QseE complex, the γ-phosphate group is transferred to a conserved His residue (H259) in QseE. Subsequent PNBM modification of this phosphate enables its detection by anti-thiophosphate ester antibody, allowing the quantification of QseE phosphorylation. The upper panel shows the phosphorylation level of QseE (p-QseE), whereas the lower panel displays the protein loading controls for both QseG and QseE. (**D**) Phosphorylation assay assessing QseE autokinase activity in the presence of QseG or small-molecule ligands. Each reaction mixture contained 10 µM QseE, 100 µM ATP-γ-S, and either 30 µM QseG or 5 mM test signaling molecules. QseE phosphorylation was detected as outlined in Fig. 1C, with QseE alone serving as the negative control. The upper panel demonstrates QseE phosphorylation levels induced by various signaling molecules, while the lower panel verifies equivalent protein loading across conditions. (**E**) Quantification of phosphorylation intensity. Phosphorylated QseE levels were normalized to the control condition (Blank, initial QseE phosphorylation without additives). Data are from *n* = 3 independent experiments, with error bars representing SEMs (ns, *P* > 0.05; *P < 0.05, unpaired two-tailed *t*-test). (**F**) eGFP fluorescence from the P_*glmY*_-*egfp* reporter under various substrate conditions. Fluorescence values were normalized to OD600. Error bars represent SEMs (*n* = 3; ns, *P* > 0.05; ***P < 0.001, unpaired two-tailed *t*-test).

The QseEF TCS is essential for the virulence of pathogenic *Enterobacteriaceae* and plays a key role in maintaining membrane homeostasis in *Escherichia coli* K-12 (26–28). Our bioinformatic analysis revealed that the *qseEF* operon is widely distributed across both pathogenic and nonpathogenic bacterial species, suggesting its involvement in diverse physiological processes (Fig. S1). Using tBLASTn searches with *qseE* and *qseF* from *Salmonella enterica* serovar Typhimurium SL1344 as references, homologous gene clusters were identified in multiple *Pseudomonadota* lineages, including *Gammaproteobacteria*, *Betaproteobacteria*, and *Acidithiobacillia*, with *qseG* consistently positioned between *qseE* and *qseF* (Fig. 1A; Fig. S1). This conserved genomic arrangement suggests that QseG likely coevolved with QseEF and plays an integral role in their signaling functions.

### QseG directly enhances QseE autokinase activity

Given the spatial colocalization of QseG and QseE (Fig. 1B) and the necessity of QseG for QseEF-mediated downstream signaling (28), we hypothesized that QseG modulates QseE kinase activity directly within the periplasm to initiate signal transduction. To test this, we examined whether QseG directly enhances QseE autophosphorylation *in vitro*. To assess the autophosphorylation level of QseE, ATP-γ-S was used as the substrate, with its γ-SP group transferred to the active histidine residue through the catalytic activity of the CA domain, resulting in the formation of a stable thiophosphohistidine. After alkylation with para-nitrobenzylmesylate (PNBM), detection was achieved using an antibody that specifically recognizes the PNBM-derivatized thiophosphate epitope (31). Similar to known TCS signal molecules (7, 32), QseG significantly and dose-dependently increased QseE kinase activity (Fig. 1C).

Sulfate, phosphate, ammonium, and catecholamines such as epinephrine and norepinephrine derived from the host have been reported as QseE activators in enterohemorrhagic *Escherichia coli* (24). Given the high sequence identity between *E. coli* and *S*. Typhimurium QseE (~87%), we assessed whether these molecules activate *S*. Typhimurium QseE *in vitro*. Sulfate, phosphate, and ammonium induced modest, statistically insignificant increases in QseE autophosphorylation, whereas epinephrine and norepinephrine had no effect (Fig. 1D, lanes 2, 4–8, and 1E, lanes 1, 3–7). Notably, QseG induced significantly higher phosphorylation than these small molecules, even at a 166.7-fold lower concentration (Fig. 1D, lane 3 and 1E, lane 2).

To assess QseG-mediated QseE activation *in vivo*, we developed a bacterial reporter system using a *glmY* promoter-driven *egfp* expression plasmid (P*_glmY_-egfp*) and an arabinose-inducible *qseG* expression plasmid (pBAD-QseG or pBAD-empty vector). Inducing *qseG* expression in *S*. Typhimurium SL1344 Δ*qseG* with 0.01% arabinose significantly increased eGFP fluorescence, as measured by a microplate reader. Consistent with *in vitro* assays, small-molecule supplementation in *S*. Typhimurium SL1344 Δ*qseG* harboring P*_glmY_-egfp* and pBAD-empty did not activate the reporter system (Fig. 1F). These findings confirm that QseG is a key modulator of QseE kinase activity.

### QseG directly interacts with dimerized QseE

**Fig 2:**
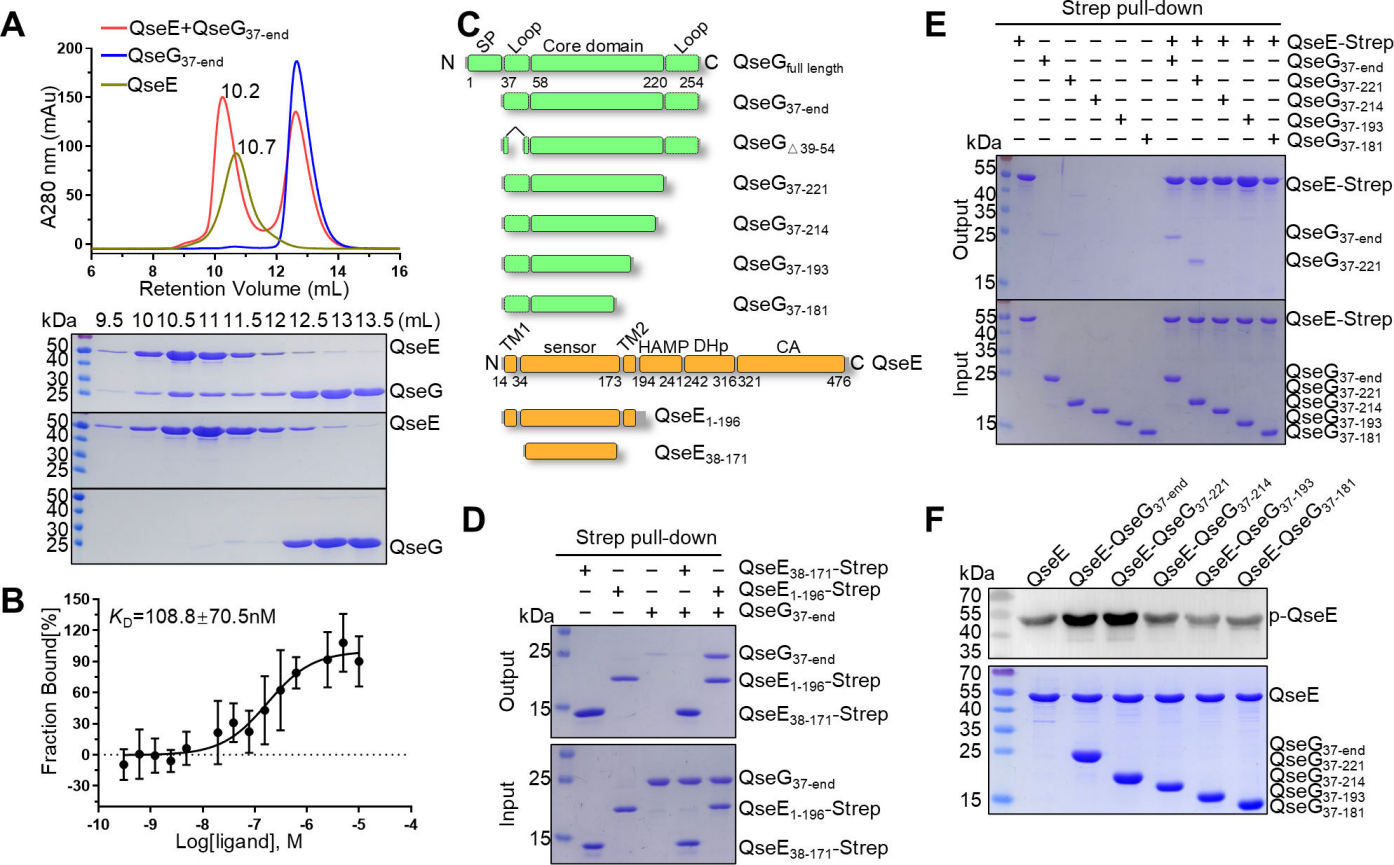
The C-terminus of outer membrane lipoprotein QseG directly interacts with dimerized QseE to activate its autokinase activity. (**A**) Elution profiles of size exclusion chromatography (Superdex 200 increase 10/300 GL) of individual recombinant QseG protein (blue) and QseE protein (brown), or of both upon incubation (red); SDS-PAGE combined with Coomassie blue staining analyses showing the protein components of the corresponding fraction 9.5 to 13.5 mL collected from the analytical size-exclusion chromatography. (**B**) Microscale thermophoresis (MST) measurement of QseG-QseE binding affinity (mean ± SEM, *n* = 3). (**C**) Schematic of the QseG and QseE variants used. Numbers refer to residues in *S*. Typhimurium SL1344 QseG and QseE. (**D**) Pull-down analysis detecting the interaction of QseG with strep-tagged QseE_1-196_ and QseE_38-171_. (**E**) Pull-down analysis of QseG truncations interacting with strep-tagged QseE. (**F**) QseE autokinase assay showing phosphorylation levels in the presence of different QseG truncations.

Bioinformatic analysis revealed that although *qseG* is co-localized with *qseE* and *qseF*, its amino acid sequence exhibits substantial divergence from *qseE* and *qseF* across bacterial taxa (Fig. 1A; Fig. S1). To investigate how QseE binds and recognizes the sequence-divergent QseG, we first examined their direct interaction *in vitro*. Gel filtration chromatography using purified QseG (mature lipoprotein, QseG_37-end_) and full-length QseE (QseE_fl_) indicated that QseG stably interacts with QseE (Fig. 2A). Microscale thermophoresis (MST) further confirmed this interaction, yielding a dissociation constant (*K*_D_) of 108  ±  70.5  nM (Fig. 2B), indicative of a relatively high binding affinity.

To further characterize the interaction mode between QseG and QseE, we designed a series of QseE truncations (Fig. 2C) and performed affinity pull-down assays using QseG_37-end_ and these strep-tagged QseE truncations. The result clearly shows that only QseE_1-196_ can bind with QseG (Fig. 2D). QseE_38-171_ represents the periplasmic sensor domain, whereas QseE_1-196_ includes both transmembrane regions and the sensor domain. Cross-linking experiments showed that QseE_38-171_ exists as a monomer in solution, whereas QseE_1-196_ and full-length QseE form dimers (Fig. S2A). Therefore, combining with the pull-down assays and cross-linking results, our findings imply that QseG binds exclusively to the dimeric form of QseE.

### The C-terminal region of QseG activates QseE autophosphorylation

The lipoprotein QseG is anchored in the outer membrane, facing the periplasm under physiological conditions, with residues 1-36 comprising its signal sequence (27). To identify the QseG region responsible for interacting with QseE, we performed pull-down assays using strep-tagged QseE to capture N- and C-terminal QseG truncations (Fig. 2C). QseG_37-end_ and QseG_37-221_ retained QseE-binding capability, whereas further C-terminal truncations (QseG_37-214_, QseG_37-193_, and QseG_37-181_) abolished this interaction (Fig. 2E). In contrast, N-terminal truncations had minimal impact on QseE binding (Fig. S2B).

To assess the physiological relevance of QseG-QseE interactions, we examined whether different QseG truncations could activate QseE autophosphorylation *in vitro*. Consistent with the pull-down results, only QseG truncations that retained QseE-binding capacity induced autophosphorylation (Fig. 2F; Fig. S2C). These findings indicate that the QseG C-terminus is essential for QseE activation, highlighting QseG’s critical role in the QseEF TCS regulatory pathway.

### Architecture of the QseG-QseE complex

**Fig 3:**
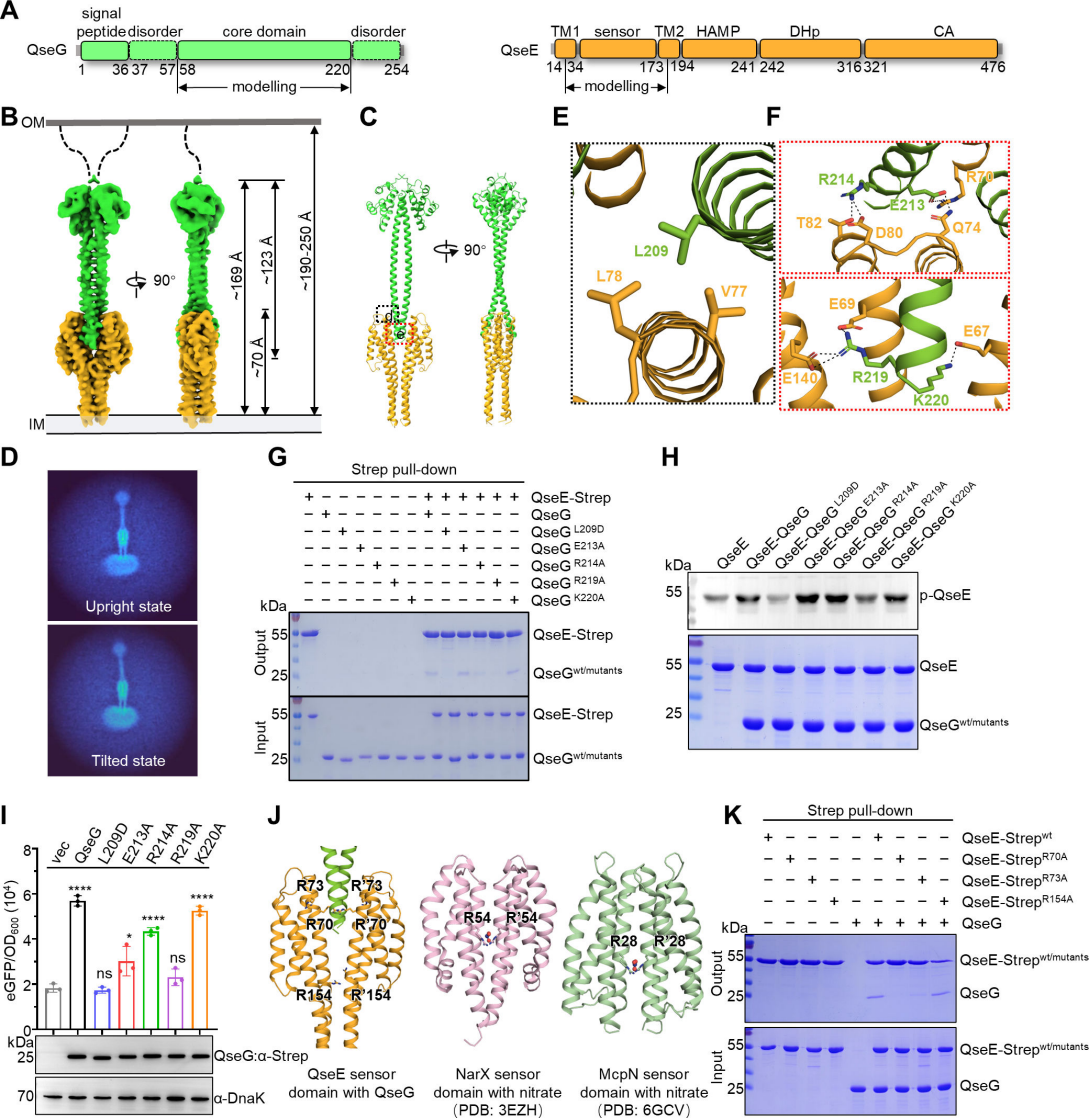
Molecular mechanism of QseG-QseE interaction. (**A**) Domain organization of *S*. Typhimurium QseG and QseE. Residue boundaries are marked, and regions used for structural modeling are annotated. (**B**) Surface view of the cryo-EM map of the QseG-QseE complex, with QseG in green and QseE in orange. Periplasmic heights are indicated. (**C**) Overall QseG-QseE structure in two orientations. (**D**) Cryo-EM classification of QseG-QseE conformations, categorizing QseG subunit orientations into upright and tilted states. (**E and F**) Enlarged views of the interaction interface, showing residues as sticks; hydrogen bonds/salt bridges are represented by dashed black lines. (**G**) Pull-down analysis of strep-tagged QseE with QseG and its mutants (L209D, E213A, R214A, R219A, and K220A). (**H**) Western blot assessing the impact of QseG and its mutants on QseE autokinase activity. (**I**) Reporter assays assessing the impact of QseG and its mutants on QseE autokinase activity. The *glmY* promoter fused to *egfp* (pSC101) was introduced into *S*. Typhimurium SL1344 *ΔqseG* cells, which were complemented with the pBAD-*qseG* plasmid and induced with 0.01% L-arabinose. OD600 and fluorescence were measured three hours post-induction. Fluorescence values were normalized to OD600. Data represent SEMs from three biological replicates (ns, *P* > 0.05; *P < 0.05; ****P < 0.0001, unpaired two-tailed *t*-test). (**J**) Structural comparison of periplasmic sensor domains in histidine kinases, showing arginine residues as sticks and substrates as ball-and-stick models. (**K**) Pull-down analysis of strep-tagged QseE and its mutants (R70A, R73A, R154A) with QseG.

To elucidate the molecular basis of QseE activation by QseG, we sought to determine the 3D structure of the QseG-QseE complex. Purified QseG_37-221_-QseE complex was successfully obtained (Fig. S3A) and subjected to cryo-EM analysis, yielding a 3.9 Å resolution map (Fig. 3A through C; Fig. S3B through G; Table S4). Due to the inherent flexibility of HK structural domains in the cytoplasm (5), the transmembrane and cytoplasmic domains of QseE—including HAMP domain (a domain found in **h**istidine kinase, **a**denylate cyclase, **m**ethyl-accepting protein, and **p**hosphatase), DHp domain (**d**imerization and **h**istidine **p**hosphotransfer), and CA domain (**c**atalytic and **A**TP-binding)—were not resolved. Notably, two-dimensional and three-dimensional classifications revealed at least two distinct conformations of the QseG-QseE complex: one in which QseG aligns upright with the QseE dimerization interface and another in which QseG adopts a tilted conformation while interacting with QseE (Fig. 3D; Fig. S3C). Such conformational variability may maintain QseG-QseE assembly under membrane stress conditions.

The final model was limited to the periplasmic region, including QseG_58-220_ and QseE_34-173_ (Fig. 3A). To refine atomic modeling, we additionally determined the crystal structures of QseG_37-221_ at 2.1 Å and the QseE sensor domain at 2.4 Å resolution (Fig. S4A; Table S5). Notably, despite the overall low resolution of the cryo-EM density maps, the QseG-QseE interface exhibited the most well-defined density, enabling accurate side-chain positioning (Fig. S4B). Structural comparison further showed that there were no significant conformational changes in the sensor domain of the QseE monomer upon QseG binding (Fig. S4A).

The complex structure reveals a C2-symmetric QseG-QseE heteromeric tetramer comprising one QseG homodimer unit and one QseE homodimer unit. The N-terminal region of each monomer QseG forms a globular fold with α-helices, whereas the C-terminus adopts a two-helix bundle, with each monomer contributing a long parallel helix. The QseE sensor domain consists of extended helices arranged in parallel and anti-parallel orientations, characteristic of the 4HB family (33–36). The C-terminal region of QseG forms a scissor-like structure that straddles the saddle-shaped dimeric sensor domains of QseE (Fig. 3C), consistent with our pull-down assay findings (Fig. 2D and E). The measured height of the complex within the periplasmic space is 169 Å (Fig. 3B). Considering that the thickness of the periplasmic space is roughly 19-25 nm (37–41) and that QseG contains an extended N-terminal loop (Cys37–Glu57), the QseG-QseE complex is likely able to span the entire periplasmic space, thereby enabling QseG to directly interact with membrane-associated QseE without detaching from the outer membrane (Fig. 3B).

Detailed structural analysis revealed that the interaction between QseG and QseE is mediated by both hydrophobic and polar interactions. Specifically, Leu209 of QseG forms hydrophobic contacts with Val77 and Leu78 of QseE (Fig. 3E). Additionally, Glu213 and Arg214 of QseG establish hydrogen bonds/salt bridges with Arg70, Gln74, Asp80, and Thr82 of QseE. Furthermore, Arg219 of QseG forms hydrogen bonds/salt bridges with Glu69 and Glu140 of QseE, whereas Lys220 of QseG interacts with Glu67 via hydrogen bonds/salt bridges (Fig. 3F).

### Key residues mediating QseG-QseE interaction and activation

To assess the functional significance of the QseG-QseE interface, we introduced site-directed mutations in key QseG residues (L209D, E213A, R214A, R219A, and K220A) predicted to be essential for complex formation. The mutant proteins were purified and examined for their ability to associate with QseE *in vitro*. Notably, the L209D and R219A mutations completely disrupted QseG-QseE complex formation (Fig. 3G, lanes 2, 9, 10, 13) and significantly reduced QseE autophosphorylation (Fig. 3H, lanes 2–4, 7 and Fig. S2D, lanes 1–3, 6). These findings highlight the crucial role of Leu209 and Arg219 in stabilizing the QseG-QseE interaction and activating QseE’s autokinase function.

To further validate the importance of Leu209 and Arg219 *in vivo*, we employed a bacterial reporter assay in *S*. Typhimurium SL1344 Δ*qseG* cells. Upon induction with 0.01% arabinose, cells expressing wild-type QseG exhibited a significant increase in eGFP fluorescence (Fig. 3I, lanes 1-2). Consistent with *in vitro* findings, the L209D and R219A mutations abolished QseEF-mediated eGFP expression (Fig. 3I, lanes 3, 6), indicating that these mutations disrupt the QseG-QseE interaction. In contrast, complementation with QseG E213A, R214A, or K220A mutants resulted in relatively high eGFP expression compared with the vector control (Fig. 3I, lanes 4–5, 7). Collectively, these results confirm that QseG enhances QseEF regulatory activity *in vivo* by directly interacting with QseE, thereby promoting its phosphorylation.

Although the sensor domain of QseE is not annotated in InterPro (42), we observed that the QseE sensor adopts a four-helix bundle (4HB) structure. Conserved arginine residues are critical for recognizing small signaling molecules, a hallmark of 4HB family sensors such as NarX (34) and McpN (33). For example, Arg54 in the sensor domain of NarX and Arg28 in McpN mediate nitrate recognition. Consistent with these observations, QseE contains two conserved arginine residues (R70 and R73) that may participate in QseG recognition (Fig. 3J). To validate this observation, we specifically constructed alanine substitution mutants targeting conserved arginine residues at the corresponding position (Fig. 3J). *In vitro* pull-down assays demonstrated that QseE R70A completely lost its ability to bind QseG, while QseE R73A displayed reduced binding, and QseE R154A exhibited no significant change in interaction compared to the wild-type (Fig. 3K). This result is consistent with our structural observations and sequence alignment analysis, which revealed that R70 and R73 are located at the interaction interface with QseG and are highly conserved across species, whereas R154 lacks both of these features (Fig. 3J and Fig. S5). This suggests that the interaction between QseE and QseG relies on a conserved signal recognition mechanism.

### The QseG-QseE interaction interface is conserved across diverse bacterial taxa

**Fig 4:**
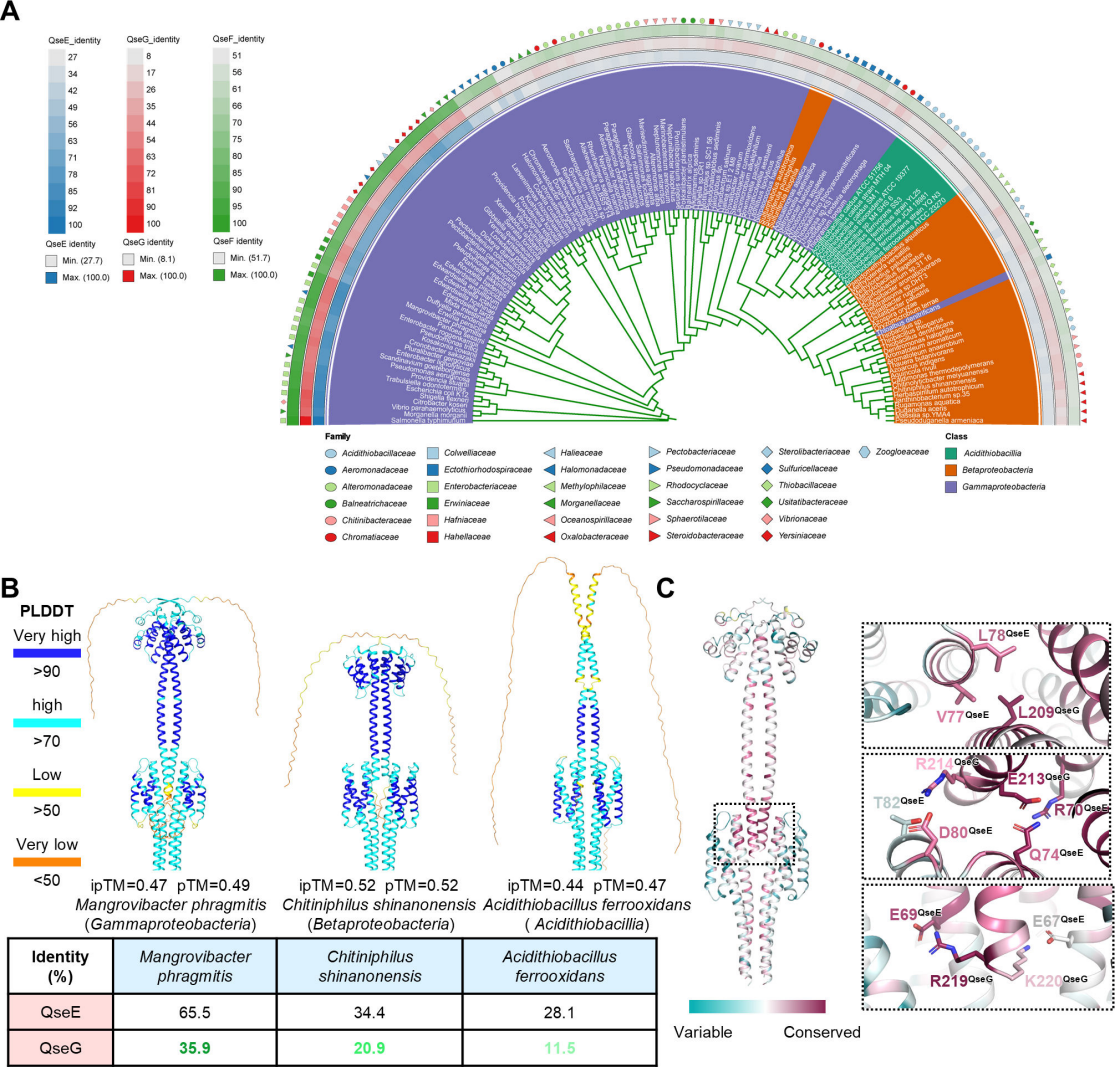
The conserved QseG-QseE interaction mode is widely distributed across *Pseudomonadota*. (**A**) Maximum-likelihood phylogenetic tree of 135 QseE-QseG-QseF homologs across *Pseudomonadota* (*Gammaproteobacteria*, *Betaproteobacteria*, and *Acidithiobacillia*). Protein sequence identities for QseE (blue), QseG (red), and QseF (green) are shown in the upper left corner and along the tree periphery. (**B**) Structural representations of QseG-QseE complexes predicted by AlphaFold3, colored by pLDDT scores (confidence in local structure). ipTM and pTM values are shown below each predicted structure. Although the ipTM scores here are below 0.6, it should be noted that the predicted ipTM for our resolved QseG-QseE complex structure is only 0.48. Protein sequence identities relative to *Salmonella* QseG and QseE are indicated. Strains are classified into three categories based on QseG amino acid sequence similarity, with color gradient intensity corresponding to different classifications. (**C**) QseG-QseE complex structure, colored by residue conservation (ConSurf analysis, left). Magnified interaction site (right), highlighting key hydrophobic and ionic interactions.

Phylogenetic analysis of the QseG-QseEF system across 31 bacterial families revealed considerable sequence divergence in each protein (Fig. 4A). To determine whether the QseE-QseG interaction is conserved across these species, we examined the structural basis of their interaction. Using AlphaFold3 (43), we predicted the QseG-QseE complex structures for three representative strains whose QseG and QseE sequences exhibited varying degrees of lower identity compared to *Salmonella*. Despite sequence divergence, these predicted structures closely resembled the resolved QseG-QseE complex (Fig. 4B). Further conservation analysis revealed that although QseG and QseE exhibit substantial sequence variability across bacterial species, the amino acid residues at their interaction interface remain highly conserved (Fig. 4C; Fig. S4C and S5). This suggests that the structural foundation of QseG-QseE interaction is preserved, maintaining QseG’s regulatory function within the QseEF two-component system. Overall, despite sequence variability among bacterial families, the interaction mechanism between the accessory lipoprotein QseG and the histidine kinase QseE remains functionally conserved.

### Outer membrane-anchored QseG is prone to engage with and activate the QseE without detaching from the outer membrane

Given the cellular localization and conserved structural features of the QseG-QseE complex, we investigated how QseG activates QseE *in vivo*. Following cleavage of the secretion signal peptide, the first N-terminal cysteine (herein, Cys37 within the long N-terminal loop_C37-E57_ of QseG in *S.* Typhimurium SL1344) undergoes lipidation, thereby anchoring QseG to the inner leaflet of the bacterial outer membrane. However, it remains unknown whether QseG must be released from the outer membrane to activate QseE.

**Fig 5:**
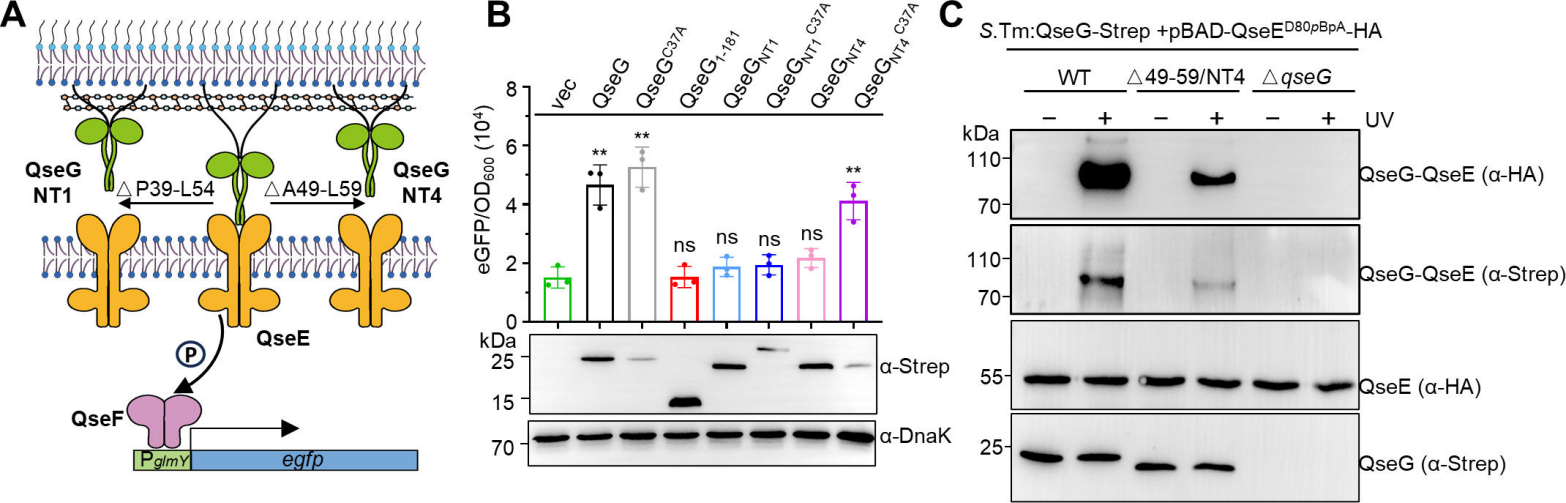
Outer membrane-anchored QseG binds to and activates QseE *in vivo*. (**A**) Schematic of eGFP protein expression controlled by the QseG-QseE-QseF three-component system. To confirm the probable persistent interaction of QseG with QseE, QseG variants NT1 and NT4 are designed using ΔP39-L54 and ΔA49-L59, respectively. (**B**) Reporter gene assay of QseG and its variants. The P*_glmY_*-*egfp* reporter plasmid and an arabinose-inducible *qseG* expression plasmid (pBAD-QseG variants or pBAD-empty vector) were co-transformed into *Salmonella enterica* serovar Typhimurium SL1344 Δ*qseG*. Reporter gene expression reflects downstream activation of QseE by the indicated QseG variants. OD600 and fluorescence were measured 3 h post-induction. Fluorescence values were normalized to OD600. An empty vector was used as a negative control. Western blot analysis of QseG and variants expression, with DnaK as a loading control. Data are representative of three independent experiments. Error bars indicate the SEMs. (ns, *P* > 0.05, not significant. **P < 0.01, unpaired two-tailed *t*-test). (**C**) Photo-crosslinking between QseG ΔA49-L59 and *p*Bpa-derived QseE. QseE(D80*p*BpA)-HA was overexpressed in wild-type *Salmonella*, QseG ΔA49-L59 mutants, and QseG knockout cells. A Strep II tag was introduced into the C-terminus of QseG and QseG ΔA49-L59 *in situ*. Cells were UV-irradiated for 10 min (+) or not irradiated (−), followed by western blot analysis with anti-HA and anti-Strep antibodies. Non-crosslinked QseG and QseE were detected with their respective tag antibodies. Molecular mass markers (kDa) are presented on the left, and the photo-crosslinked QseE-QseG products are detected at the 80 kDa position.

To assess it, we evaluated the function of two QseG N-terminal truncations (NT1 and NT4) and their respective C37A variants through reporter-gene assays. Mutation of the lipidation site (Cys37) to alanine prevents membrane anchoring, resulting in predominantly periplasmic localization of QseG (44). The NT1 variant contains a 16-residue deletion (Pro39-Leu54), whereas NT4 features an 11-residue deletion (Ala49-Leu59) (Fig. 5A). Subsequent *in vitro* pull-down assays with purified proteins revealed that all truncated variants (NT1/NT4) and their C37A derivatives (NT1^C37A^/NT4^C37A^) maintained wild-type-level QseE binding capacity (Fig. S6A). Furthermore, autokinase activity assays demonstrated these variants preserved the ability to enhance QseE autophosphorylation with efficiency comparable with wild-type QseG (Fig. S6B).

However, compared with the Δ*qseG* mutant complemented with full-length QseG, cells expressing NT1 or NT4 failed to activate *egfp* expression (Fig. 5B, lanes 1, 2, 5, and 7), likely due to their inability to reach QseE while anchored in the outer membrane. Consistent with our hypothesis, NT4^C37A^, which was released into the periplasm, successfully restored *egfp* activation (Fig. 5B, lanes 3, 8). In contrast, NT1^C37A^ did not recover activity, possibly due to impaired signal peptidase cleavage (Fig. 5B, lane 6 and middle panel). These findings indicate that the outer membrane-anchored QseG directly interacts with and activates QseE *in vivo*, rather than being released into the periplasm.

We further performed an *in vivo* site-specific photocrosslinking experiment by incorporating *p*-benzoyl-L-phenylalanine (*p*Bpa) at position Asp80 of HA-tagged QseE in *S.* Typhimurium SL1344, NT4, and Δ*qseG* strains. QseG was carried to a C-terminal Strep tag, and cells were cultivated under normal conditions before UV exposure. Following UV treatment, cross-linked QseG-QseE bands (~80 kDa) were detected in cells expressing QseG using anti-Strep (detecting QseG) and anti-HA (detecting QseE) antibodies (Fig. 5C, lane 2, upper and middle panels). These bands were faint in NT4-expressing cells (Fig. 5C, lane 4, upper and middle panels) and absent in the Δ*qseG* control. Together, these findings confirm that the outer membrane-anchored QseG is prone to interact with and activate QseE under standard culture conditions.

## DISCUSSION

**Fig 6:**
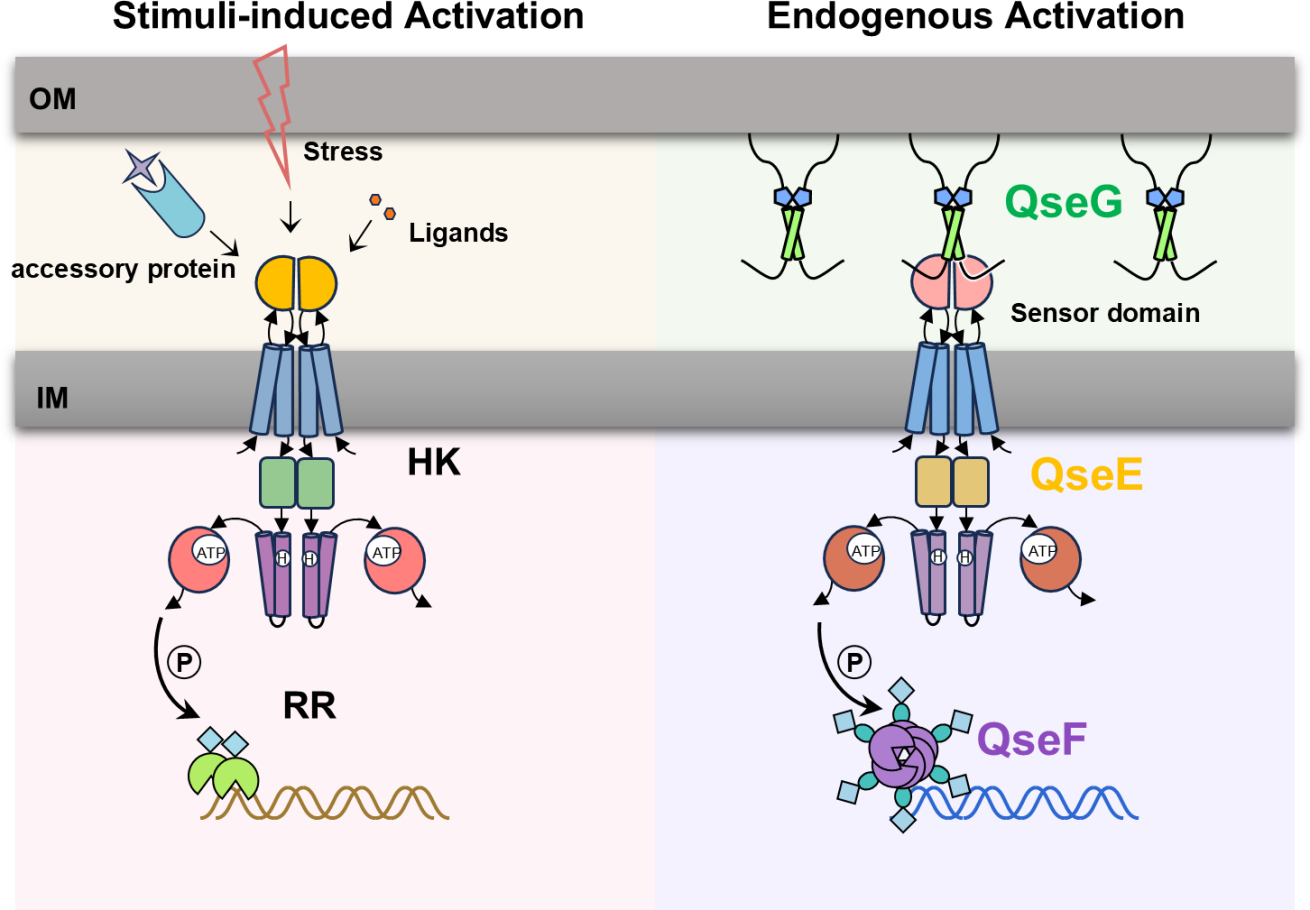
Schematic model proposed for signal perception through QseGE. In the typical signal transduction pathway, histidine kinase (HK) is activated via sensing external stress, binding signal molecules, or stimulation by auxiliary proteins. Once activated, HK transfers a phosphate group to the response regulator (RR), triggering downstream gene expression regulation (left panel). Under standard culture conditions, QseG is anchored to the outer membrane and is an endogenous protein activator, persistently interacting with QseE (HK). This interaction keeps QseE in a constitutively active state (right panel).

Although the downstream functions of two-component systems (TCSs) have been widely explored, the mechanisms underlying signal sensing and regulation mediated by accessory proteins remain poorly understood. In fact, the majority of available evidence to date indicates that activation of inner membrane-localized histidine kinases requires either direct binding to environmental signals or mediation by accessory proteins that are embedded in the inner membrane, localized in the periplasmic space, or released from the outer membrane (Fig. 6, left panel). In contrast, here, we systematically characterized the outer membrane lipoprotein QseG as an endogenous activator of the QseEF TCS in *S.* Typhimurium, demonstrating that it is prone to engage with and promotes QseE activation without detaching from the outer membrane (Fig. 6, right panel). This discovery provides new insights into signal perception and transmission from the outer to the inner membrane, unveiling a previously unrecognized mechanism of accessory protein–histidine kinase (HK) interaction in TCS activation.

QseG is anchored within the inner leaflet of the outer membrane, whereas histidine kinases mostly localize to the inner membrane. The peptidoglycan layer separates the periplasm into two distinct compartments, raising the intriguing question of how signals directly associated with the outer membrane can be transmitted to the inner membrane-bound HK. Our findings suggest that QseG bridges the outer and inner membranes, facilitating the transduction of signals into the cytoplasm. Similar but different membrane-spanning mechanisms have been described for accessory lipoproteins such as NlpE and RcsF, which regulate bacterial envelope stress responses (21, 22). NlpE serves as a sensor enabling the Cpx system to monitor lipoprotein trafficking efficiency. Upon trafficking defects, NlpE—normally localized to the outer membrane—mislocalizes to the inner membrane, where it aberrantly interacts with CpxA, thereby activating the downstream Cpx pathway (19, 30, 44–46). Additionally, RcsF responds to outer membrane perturbations by interacting with the inner membrane protein IgaA, alleviating its repression of the RcsCDB phosphorelay, which regulates stress-adaptive responses such as biofilm formation and capsular polysaccharide biosynthesis (13, 21). Hence, QseG-mediated activation of QseE represents a distinct regulatory model compared to the NlpE-CpxA and RcsF-RcsCDB pathways.

The unexpectedly high binding affinity between QseG and QseE (*K*_D_ = 108 ± 70.5 nM) is substantially stronger than typical transient ligand-HK interactions, such as CitA-citrate (*K*_D_ = 5.5 µM)(47), suggesting that QseG forms a highly stable complex with QseE. Given the stoichiometric excess of QseG over QseE (Fig. S6C), their interaction probably facilitates basal activation rather than stimulus-dependent regulation. This is consistent with the previously mentioned notion that the QseEF system predominantly exists in an “on” state (28), as well as with our current findings (Fig. 5). In canonical TCSs, feedback mechanisms typically reset phosphorylation states to enable dynamic signal modulation (12). However, the QseEGF system lacks a clear negative regulatory component, raising the question of how signal termination occurs. Despite extensive efforts, the physiological mechanism that disrupts QseG-QseE interaction remains unclear. We propose two potential models: (i) mechanical disruption due to periplasmic expansion or outer membrane damage, or (ii) stress-induced regulation via periplasmic proteolysis of QseG or QseE. Further investigations are required to elucidate whether cellular feedback mechanisms transiently suppress the QseEGF pathway in specific contexts.

A previous study has reported that in *E. coli* K12, the Ser58 mutation in the periplasmic domain of QseE impaired QseG-mediated activation of QseE by partially disrupting their interaction (28). However, our cryo-EM structural analysis of the *Salmonella* QseG-QseE complex reveals that Ser58 does not directly bind to QseG (Fig. S4D). Sequence alignment further supports this, revealing poor conservation of Ser58 across species compared with the conserved interaction interface (Fig. S5B). We speculate that the S58N mutation may subtly affect QseE dimerization, indirectly impacting QseG-QseE interaction via allosteric effects. The detailed structural insights obtained in this study provide robust evidence that the primary interaction interface between QseG and QseE differs from previously proposed models involving Ser58 (28).

Additionally, a recently published crystal structure of the *Escherichia coli* QseG and QseE periplasmic domain proposed a potential interaction model, postulating an interaction between the positively charged surface of QseE and a negatively charged region on the C-terminal helical extension (α7c) of QseG (36). However, our cryo-EM structure of the QseG-QseE complex, corroborated by mutational analysis, unequivocally demonstrates that the hydrophobic interface mediated by residue L209 is essential for this interaction. The detailed structural insights obtained in this study provide robust evidence that the primary interaction interface between QseG and QseE differs from previously proposed models (28, 36).

Owing to the inherent flexibility and dynamics of histidine kinases (5, 10, 48), the structure of a full-length histidine kinase has yet to be determined. In our study, despite using full-length QseE to assemble the QseG-QseE complex for cryo-EM analysis, we were unable to resolve the full-length structure, with the intracellular domain remaining elusive. This limitation hindered a structural interpretation of the conformational changes in QseE upon QseG binding. Previous studies on EnvZ have demonstrated that upon sensing increased osmolarity (induced by salt or sugars) or decreased pH, the backbone of a helical bundle within the DHp domain becomes stabilized. The conserved histidine residue His243, essential for autophosphorylation, is located within this helix. Enhanced helical backbone stabilization weakens interactions between adjacent residues (Ala239 and Asp244) and the catalytic His243, releasing structural constraints and enabling conformational changes that facilitate its phosphorylation and subsequent binding to the response regulator OmpR, thereby enhancing phosphotransfer and downstream signaling (49–53). In light of this theory, we propose that the conformational change triggered by QseG binding is transmitted through the transmembrane helices, leading to stabilization of the helical bundle containing His259 in the DHp domain of QseE in *S*. Typhimurium. This stabilization is predicted to alleviate conformational constraints around His259, rendering it more susceptible to autophosphorylation and enhancing its binding affinity for the response regulator QseF. Subsequent phosphorylation of QseF is anticipated to promote its oligomerization, facilitating recognition and binding to promoter regions of target genes, thereby initiating transcriptional activation. Therefore, from this perspective, the QseG-QseE-QseF signaling pathway can be classified as a three-component system. Further work is needed to elucidate the underlying process.

In summary, our study identifies a novel regulatory model in which the outer membrane-anchored lipoprotein QseG sufficiently activates the QseEF TCS. Together with prior findings on this important TCS (24–27), these results imply that targeting the QseG-QseE interaction may represent a potential strategy for mitigating bacterial virulence and infection.

## MATERIALS AND METHODS

### Bacterial strains and growth conditions

The bacterial strains utilized in this investigation are comprehensively detailed in Table S1. All *Salmonella enterica* serovar Typhimurium strains were derived from the parental wild-type strain SL1344. Targeted gene deletions were generated via double-crossover homologous recombination employing the pSB890 (tetR) integration vector system. All *Salmonella* strains and *E. coli* strains were routinely propagated in lysogeny broth (LB) medium (BD Biosciences) at 37°C under aerobic conditions with 200 rpm orbital shaking. Antibiotic supplementation was implemented as required at the following concentrations: kanamycin (50 µg/mL), ampicillin (100 µg/mL), tetracycline (10 µg/mL), and spectinomycin (50 µg/mL).

### Plasmid construction

The plasmid constructs generated in this study, along with the primers involved, are cataloged in Tables S2 and S3, respectively. For recombinant protein expression in *E. coli*, QseG truncation mutants (QseG_37-end_, QseG_37-221_, QseG_37-214_, QseG_37-193_, QseG_37-181_) were subcloned into the pET22b expression vector. All constructs incorporated an N-terminal pelB signal peptide to direct periplasmic localization, followed by a Strep II affinity tag and tobacco etch virus (TEV) protease recognition site. The full-length QseE coding sequence (QseE_fl_) was PCR-amplified from *S*. Typhimurium SL1344 genomic DNA and directionally cloned into the pET28b vector using the Gibson assembly strategy, resulting in a C-terminal Strep II tag fusion. Additional constructs (QseE_38-171_, QseE_1-196_, QseE-eGFP) were engineered into pET28b expression vectors containing an N-terminal hexahistidine-SUMO (small ubiquitin-like modifier) tag for enhanced solubility.

For the construction of eGFP reporter strains, a dual-plasmid system was constructed combining pSC101 and pBAD (arabinose-inducible) vectors. The *egfp* reporter gene, driven by the native *glmY* promoter, was inserted into the pSC101 vector to generate the pSC101-P*_glmY_-egfp* construct. The *qseG* gene was inserted into the pBAD vector downstream of the araBAD promoter, creating pBAD-*qseG* expression plasmids.

To investigate QseG-QseE interactions through photo-crosslinking, we implemented a bipartite expression system consisting of pUltra and pBAD plasmids. The pUltra plasmid was reconfigured to co-express orthogonal tRNA/aminoacyl-tRNA synthetase (aaRS) pairs under arabinose-inducible control. Concurrently, the pBAD plasmid was constructed to express a QseE variant with an amber codon substitution at position D80 for the incorporation of the noncanonical amino acid *p*Bpa (QseE D80*p*Bpa), fused to a C-terminal HA epitope tag. Native QseG expression was maintained chromosomally as a C-terminal Strep-tag II fusion.

### Protein expression and purification

For recombinant protein expression, *E. coli* C43(DE3) cells were transformed with the expression plasmids described above and grown in LB medium to OD_600_  =  0.8 (for QseE, OD_600_ = 1.2). Protein production was induced with 0.2  mM isopropyl-β-d-thiogalactopyranoside (IPTG) followed by 16 h incubation at 20°C. Cell pellets were harvested by centrifugation (4,500 × *g*, 15 min, 4°C) and resuspended in lysis buffer (20 mM Tris-HCl, pH 8.0, and 150 mM NaCl).

For the purification of QseG, cells were lysed using a high-pressure homogenizer (Union-Biotech, China), and the supernatant was separated from the pellet by ultracentrifugation (17,000 × *g*, 60 min, 4°C). The clarified supernatant was loaded onto a gravity-flow chromatography column (Bio-Rad) packed with 2  mL of Strep-Tactin agarose resin (Smart-Lifesciences, China) pre-equilibrated with 20  mL of lysis buffer. After sequential washing with five column volumes of lysis buffer, bound proteins were subjected to on-column TEV protease cleavage (100 µg/mL) for 2 h at 25°C. Eluted fractions were further purified by size-exclusion chromatography (SEC) using a Superdex 200 Increase 10/300 GL column (Cytiva) pre-equilibrated with lysis buffer. Target protein fractions were concentrated to 20 mg/mL using 30 kDa MWCO centrifugal filters (Millipore), with the final purity assessed by both UV-Vis spectral analysis and SDS-PAGE.

Membrane-bound QseE purification required differential centrifugation following cell disruption. After initial clarification (17,000 × *g*, 20 min), membrane fractions were isolated by ultracentrifugation (150,000 × *g*, 60 min) and solubilized in lysis buffer containing 1% (wt/vol) lauryl maltose neopentyl glycol (LMNG; Anatrace) and 0.1% (wt/vol) cholesteryl hemisuccinate (CHS; Anatrace) through gentle agitation (4°C, 2 h). After centrifugation at 150,000 × *g* for 30 min, detergent-solubilized proteins were affinity-purified using Strep-Tactin agarose resin with wash/elution buffers containing optimized detergent concentrations (0.002% LMNG, 0.0002% CHS). Final polishing was achieved through SEC in a detergent-containing buffer using a Superdex 200 Increase column.

To obtain the QseG-QseE complex, QseG_37-221_ and QseE proteins were mixed at a 3:1 molar ratio and then incubated with gentle rotation at room temperature for 30 min. The complex was then separated using SEC and concentrated to 4 mg/mL using 100 kDa MWCO centrifugal filters (Millipore) for cryo-electron microscopy studies.

### Crystallization, data collection, and structure determination crystallization

The QseG_37-221_ crystals grew at 18°C using the hanging-drop vapor-diffusion method in a mix of 1 µL of protein with 1 μL of reservoir solution consisting of 15% (wt/vol) PEG8000, 0.1 M sodium cacodylate, pH 6.7, 0.2 M magnesium acetate. Similarly, QseE_38-171_ crystals grew out of a well containing 3.5 M Sodium formate, 0.1 M Tris-HCl, pH 8.5. Crystals were cryo-protected in reservoir solution supplemented with 25% (vol/vol) glycerol and flash-cooled in liquid nitrogen for data collection. Data sets were collected under cryogenic conditions (100K) on BL19U beamline at the Shanghai Synchrotron Radiation Facility (Shanghai, China). All data were processed using HKL3000 (54), and molecular replacement was carried out using PHENIX (55). Initial models for molecular replacement were generated using AlphaFold2 (56). Structure refinement was carried out in PHENIX, alternated with manual fitting in Coot (57). Data collection and structure refinement statistics are included in Table S5.

### Cryo-EM sample preparation and data collection

In total, 4  µL of sample was applied to freshly glow-discharged Quantifoil R1.2/1.3 Au 300 mesh grids, followed by blotting for 3.5 s at 100% humidity and plunge-freezing in liquid ethane using a Vitrobot Mark IV system (Thermo Fisher Scientific). The qualified grids were loaded into a Titan Krios G4 transmission electron microscope (Thermo Fisher Scientific) operating at 300 kV with a K3 BioQuantum direct electron detector (Gatan) and GIF Quantum LS energy filter (20 eV slit width). Data acquisition was performed in counting mode at 81,000× nominal magnification (calibrated pixel size 0.53 Å/pixel) under low-dose conditions (50 e-/Å² total dose) using Leginon v4.12 automation. Each movie stack comprised 40 frames with 0.08 s frame exposure time (3.2 s total exposure). Frame alignment and dose compensation were implemented through MotionCor2 v1.6.0 using a B-factor of 150 for weighted averaging, followed by 2 × Fourier binning to 1.06 Å/pixel.

### Image processing and 3D reconstruction

For the QseG_37-221_-QseE complex data set, 5,560 micrographs were collected. The image processing steps were carried out using cryoSPARC (58). All micrographs were imported, and the CTF corrections were performed using Patch CTF estimation. After the deletion of bad micrographs, 300 micrographs were used for automatic picking by blob picker, and these particles were subjected to 2D classification. The class averages representing projections of the QseG-QseE complex in different orientations were chosen as templates for template picking from the whole data set. A total of 8,488,000 particles were picked from 5,238 micrographs. These particles were extracted and binned four times and subjected to 2D classification. After three rounds of 2D classification, ~1,626K particles in good 2D averages were chosen and subjected to the ab initio reconstruction and the following heterogeneous refinement. The map of each class was measured in UCSF Chimera (59), and the particles from good classes were selected and re-extrated to the original pixel size of 1.06 Å. After another round of ab initio reconstruction and heterogeneous refinement to further remove bad particles, the best 88,834 particles were used for 3D reconstruction by non-uniform (NU) refinement (60) to yield a map at 3.9 Å resolution with C2 symmetry imposed. The local resolution map was calculated using local resolution estimation in cryoSPARC and displayed in Chimera X (61).

For the initial construction of the model of the QseG-QseE complex, we relied on the structural blueprints provided by the crystal structure of QseG_37-221_ (PDB: 9M06) and QseE_38-171_ (PDB: 9M07). These structures were computationally docked into an electron microscopy density map using UCSF Chimera and manually adjusted in Coot (57), followed by refinement using Phenix (62) in real space with secondary structure and geometry restraints to prevent structure overfitting. Statistics of 3D reconstruction and model refinement are summarized in Table S4.

### Pull-down assay

Strep-tagged QseE, along with QseG truncation mutants and point mutants, was expressed in *E. coli* C43 (DE3). Protein purification was performed as described above. As designed, the purified Strep-tagged QseE (20 µM) and the QseG (40 µM) mutations were mixed in 200  µL binding buffer (20  mM Tris-HCl, pH 8.0, 150  mM NaCl, 0.002% LMNG, and 0.0002% CHS). Then, 20  µL Strep-Tactin resin beads (Smart-Lifescience, China) were added and incubated at 25°C for 30 min. After the beads were extensively washed three times with binding buffer, the remaining bound proteins were eluted with 30 µL elution buffer (5 mM D-Desthiobiotin, 20  mM Tris-HCl, pH 8.0, 150  mM NaCl, 0.002% LMNG, and 0.0002% CHS). The eluted samples were detected by SDS-PAGE and stained with Coomassie blue. Negative controls with single-protein samples confirmed assay specificity.

### Analytical size-exclusion chromatography

The size-exclusion chromatography (SEC) analyses were performed using an AKTA FPLC system (GE Healthcare) with absorbance monitored at 280 nm. Individual protein samples and mixed protein samples (in a 1:2 molar ratio of QseE to QseG) were prepared in a volume of 500 µL. Prior to loading, mixed protein samples were incubated at 37°C for 30 min. The samples were then loaded onto a Superdex 200 10/300 GL column, both pre-equilibrated with the same column buffer used in QseE protein purification. The resulting chromatographic data were analyzed and fitted using GraphPad Prism v.8.0 software, with the outputs aligned for comparative analysis.

### Microscale thermophoresis (MST)

For MST measurements, the QseE-eGFP protein and QseG_37-end_ were purified from *E. coli*, as described above. All samples were dialyzed into the same MST assay buffer containing 20 mM HEPES, pH 7.5, 150  mM NaCl, 0.02% n-dodecyl-β-D-maltopyranoside (DDM), and 0.05% Tween 20. QseG_37-end_ with serial dilution was mixed with QseE-eGFP. The mixture was incubated in the darkroom at 25°C for 30  min before measurement. Capillaries were then filled with samples individually and loaded into the Monolith NT.115 (Nanotemper Technologies) instrument with the following thermophoresis parameters: medium MST power and 40% LED. The MST data were analyzed with MO Affinity Analysis software.

### Autokinase assay

The phosphorylation level of HK was assessed using ATP-γ-S (abcam, ab138911) and a primary antibody that specifically recognizes alkylated thiophosphate esters, which were modified by the alkylation reagent PNBM (abcam, ab138910) (31). QseE (10 µM) was incubated with varying concentrations of QseG (30 µM) or other substrate molecules (5 mM) for 10 min at room temperature. Subsequently, the mixture of 10 µM QseE and the respective molecules was further incubated with 100 µM ATP-γ-S in a kinase reaction buffer (20 mM Tris-HCl, pH 7.5, 100 mM NaCl, 5 mM KCl, 5 mM MgCl_2_, 5% glycerol, 0.002%LMNG, and 0.0002% CHS) for 30 min at 37°C. The reaction was terminated by the addition of 20 mM EDTA. Thiophosphorylated histidine was then alkylated with 2.5 mM PNBM for 1 h at RT. The phosphorylated QseE was separated by 12% SDS-PAGE and then transferred to a polyvinylidene difluoride (PVDF) membrane at a constant current of 180 mA for 35 min at 4°C using transfer buffer (20 mM Tris, pH 8.3, 192 mM glycine, 10% [vol/vol] ethanol). The PVDF membrane was blocked with 5% nonfat milk in TBST buffer (20 mM Tris, pH 7.6, 150 mM NaCl, 0.05% [vol/vol] Tween-20) and probed with a monoclonal anti-thiophosphate ester antibody (Abcam, ab92570) at 1:5,000, which was detected with anti-rabbit HRP antibody (MBL, 458) at 1:5,000. The band intensities of phosphorylated QseE were quantified using ImageJ software and normalized to the intensity observed in the control (CK) group.

### Sequence alignment and phylogenetic analysis

To identify QseEGF homologs in bacterial genomes, the amino acid sequence of QseEF from *S. enterica* serovar Typhimurium strain SL1344 was used to query the protein database composed of the whole-genome sequences in the NCBI genome database using tBLASTn. Initial screening identified 5,000 putative QseE homologs spanning diverse bacterial taxa. To ensure phylogenetic robustness, we implemented a rigorous filtering pipeline: removal of redundant strains; exclusion of genomes lacking operon-encoded QseG/QseF homologs; manual verification of tripartite system integrity. This yielded 135 non-redundant, phylogenetically diverse QseEGF systems for subsequent analyses.

The protein sequences were fused in the order of QseE, QseG, and QseF. Multiple sequence alignment of the representative fusion proteins was performed with Clustal Omega, retaining conserved alignment blocks. A maximum likelihood phylogenetic (MLP) tree was constructed with IQ-TREE following automatic model selection, and node support was assessed using 1,000 ultrafast bootstrap replicates. In the phylogenetic tree, QseE, QseG, and QseF are represented by blue, red, and green blocks, respectively, with color gradients reflecting their similarity to the corresponding *Salmonella* proteins. Different bacterial families are denoted by distinct color blocks on the outermost ring of the phylogenetic tree.

### *In vivo* photo-crosslinking

The *in vivo* photo-crosslinking assay was performed according to the published method with optimized modifications. *S. enterica* serovar Typhimurium SL1344 mutant strains with the *qseG* fused to a C-terminal strep II tag co-transformed with pUltra-aaRS-tRNA and pBAD-*qseE*-HA (D80*p*Bpa) were cultured in 5 mL of LB medium supplemented with 50 µg/mL spectinomycin and 50 µg/mL ampicillin under aerobic conditions at 37°C overnight. Primary cultures were diluted 1:100 into 50 mL fresh LB medium containing 1 mM *p*-benzoyl-L-phenylalanine (*p*Bpa) and 0.2% arabinose. The cultures were incubated at 37°C with agitation to an OD_600_ of 0.8. After expression, the cell pellets were harvested and suspended in phosphate-buffered saline (PBS, pH 7.4). Half of the culture (25  mL) was irradiated with UV light at 365 nm for 10 min, whereas the other half was untreated. Subsequently to lysis via sonication, HA-tagged QseE was extracted with 1% (wt/vol) DDM in TBS buffer (50 mM Tris-HCl, 150 mM NaCl, pH 7.5). Then, the supernatant was incubated with Strep tactin resin. After washing away non-specific proteins, the target protein was eluted for western blot detection.

### Reporter-gene assay

Plasmid pSC101, which contains an *egfp* gene on a *glmY* promoter, was co-transformed with a pBAD vector expressing various QseG mutants into *S. enterica* serovar Typhimurium SL1344 *qseG* gene knockout strains. Other small molecules (5 mM) were added to the *qseG* knockout strains that were transfected with both the pSC101-P*_glmY_-egfp* plasmid and the pBAD vector. The reporter construction strains were cultured overnight in LB medium supplemented with kanamycin (50 µg/mL) and ampicillin (100 µg/mL) and then diluted 1:100 to fresh medium supplemented with 0.01% arabinose and cultured at 37°C for 3 h. Subsequently, 100 µL culture was washed three times with PBS buffer before being added to a 96-well plate to measure eGFP fluorescence and OD_600_. Each experimental condition was performed in triplicate, and the fluorescence intensity of each well was normalized by dividing the absolute fluorescence value by the relative bacterial concentration of each group, with the wild type serving as the reference (normalized to 1). Three independent biological replicates were conducted with randomized sample loading.

### Statistical analysis

All statistical analyses were calculated using GraphPad Prism v.8.0 (GraphPad). For comparisons of the means of two groups, unpaired two-tailed *t*-tests were used. The significance of mean comparison was annotated as follows: ns, not significant; *, *P* < 0.05; **, *P* < 0.01; ***, *P* < 0.001. *P* < 0.05 was considered statistically significant.

## Data Availability

The atomic coordinates and structure factors generated in this study have been deposited in the Protein Data Bank (PDB) under the accession code: 9M06, 9M07, and 9M08. Cryo-EM maps have been deposited to the Electron Microscopy Data Bank (EMDB) with accession numbers: EMD-63539.

## SUPPLEMENTAL MATERIAL

Supplemental material mbio.02864-25-s0001.pdf Supplemental figures and tables

ASM does not own the copyrights to Supplemental Material that may be linked to, or accessed through, an article. The authors have granted ASM a non-exclusive, world-wide license to publish the Supplemental Material files. Please contact the corresponding author directly for reuse.
