# Supplementary material for: Cryo-EM structure of the QseG-QseE complex reveals an accessory protein-driven two-component system activation mechanism
Source: mBio. 2025 Nov 17;16(12):e02864-25. doi: 10.1128/mbio.02864-25 (PMC12691602; doi:10.1128/mbio.02864-25)
Supplement: Supplemental material — Supplemental figures and tables [file mbio.02864-25-s0001.pdf]

Cryo-EM structure of the QseG-QseE complex reveals an accessory protein-driven two-component system activation mechanism

Piqian Gong[1], Guobang Li[1,2,*], Weixun Li[1], Mengyuan Xu[1], Xuyao Jiao[1], Xudong Chen[3], Beile Gao[4], Xiang Gao[1,5,*]

[1] State Key Laboratory of Microbial Technology, Shandong University, Qingdao 266237, China

[2] Institutes of Biomedical Sciences, College of Life Sciences, Inner Mongolia University, Hohhot 010021, China

[3] Ministry of Education Key Laboratory of Protein Science, Tsinghua-Peking Center for Life Sciences, Beijing Advanced Innovation Center for Structural Biology, School of Life Sciences, Tsinghua University, Beijing 100084, China

[4] State Key Laboratory of Tropical Oceanography, Guangdong Provincial Key Laboratory of Applied Marine Biology, South China Sea Institute of Oceanology, Chinese Academy of Sciences, Guangzhou, 510301, China

*E-mail: gbli@imu.edu.cn (G.B.L.), xgao@email.sdu.edu.cn (X.G.)

Supplemental Figures and Tables

Page 3: **Supplemental Figure S1**. The *qseEGF* gene cluster is widespread across *Pseudomonadota*.

Page 4: **Supplemental Figure S2**. QseG interacts with dimerized QseE through its C-terminus.

Page 5: **Supplemental Figure S3**. Structure determination of $QseG_{37\text{-}221}$-$QseE_{FL}$ complex by cryo-EM.

Page 6: **Supplemental Figure S4**. Structural insights into the QseG–QseE interaction interface.

Page 7-11: **Supplemental Figure S5**. Multiple sequence alignment of QseG and QseE reveals conserved residues.

Page 12: **Supplemental Figure S6**. Pull-down analysis and autokinase assay of QseG N-terminal mutations and the western blot characterization of the relative expression level of QseG and QseE in Salmonella typhimurium SL1344.

Page 13: **Supplemental Table S1**. Bacterial strains used in this study.

Page 14: **Supplemental Table S2**. Plasmid used in this study.

Page 15-16: **Supplemental Table S3**. List of oligonucleotides used in this study.

Page 17: **Supplemental Table S4**. Cryo-EM data collection, refinement and validation statistics.

Page 18: **Supplemental Table S5**. QseG and QseE crystal data processing and structure refinement statistics

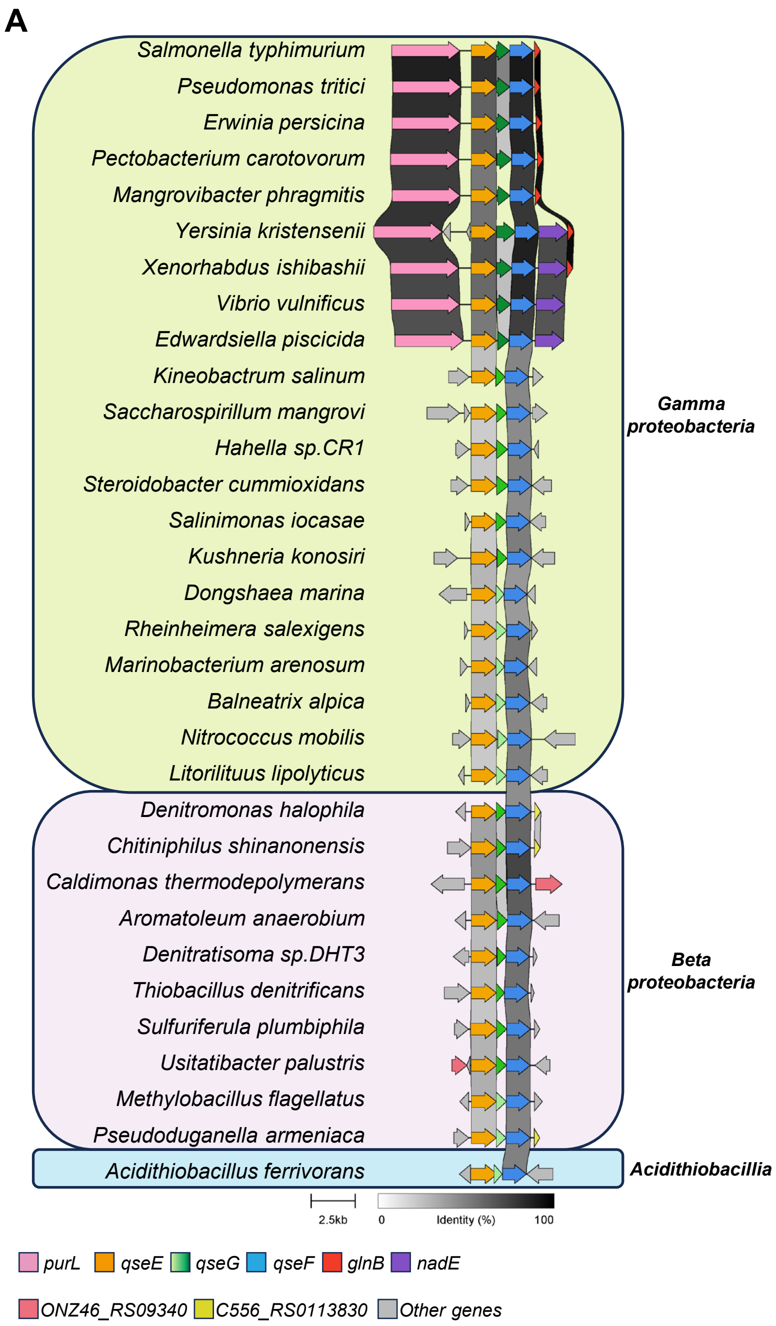

Supplemental Figure S1. The *qseEGF* gene cluster is widespread across *Pseudomonadota.*
(**A**) Alignments of gene loci containing *qseEGF* genes from strains belonging to different bacterial families. Homologous genes are depicted with the same colors and are connected by shading in grayscale, with intensity reflecting the amino acid sequence identity. Predicted functions of ORFs in gene loci are represented by different color blocks. Based on bacterial taxonomy, these families are distributed across three distinct classes within the phylum *Proteobacteria*: *Gammaproteobacteria*, *Betaproteobacteria*, and *Acidithiobacillia*. ORFs with different predicted functions are color-coded: *purL* (pink), *qseE* (orange), *qseG* (gradient green), *qseF* (blue), *glnB* (red), *nadE*(purple) and non-conserved genes (gray).
.

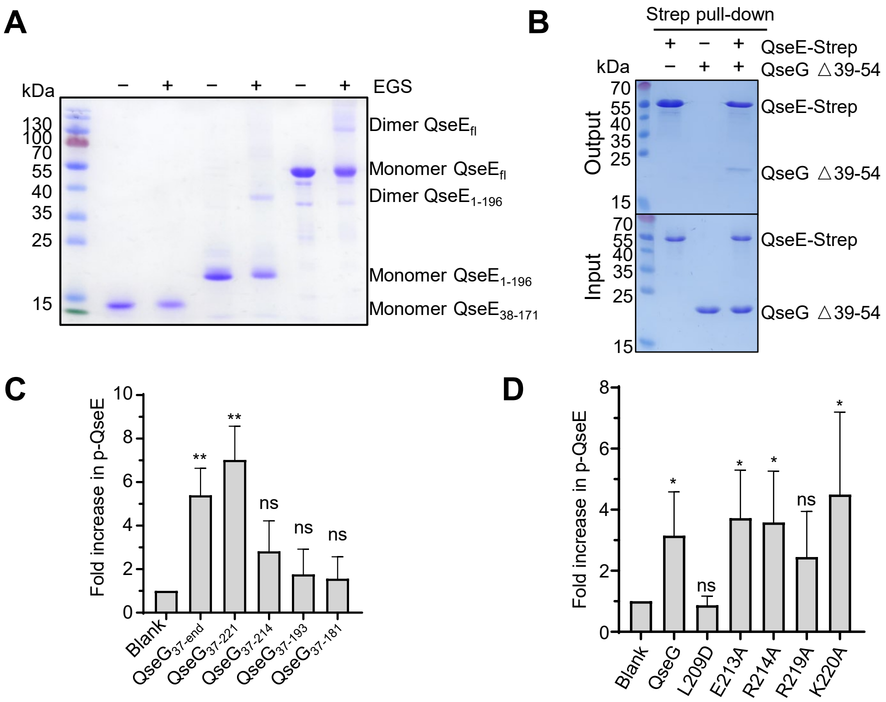

Supplemental Figure S2. QseG interacts with dimerized QseE through its C-terminus.

(**A**) Purified QseE$_{fl}$, QseE$_{1-196}$, and QseE$_{38-171}$ were incubated with or without the EGS crosslinker and detected using Coomassie Brilliant Blue. The monomer and dimer protein bands are marked.

(**B**) Pull-down analysis to detect the interaction of Strep-tagged QseE with the N-terminally truncated QseG variant QseG△39-54.

(**C**-**D**)Western blot assessing the impact of QseG wild-type, truncations (**C**) and mutants (**D**) on QseE autokinase activity. Phosphorylation intensity quantification using ImageJ, normalized to the Blank control. Data represent SEMs from three independent experiments (ns, $P > 0.05$, not significant. *$P < 0.05$, **$P < 0.01$, unpaired two-tailed *t*-test).

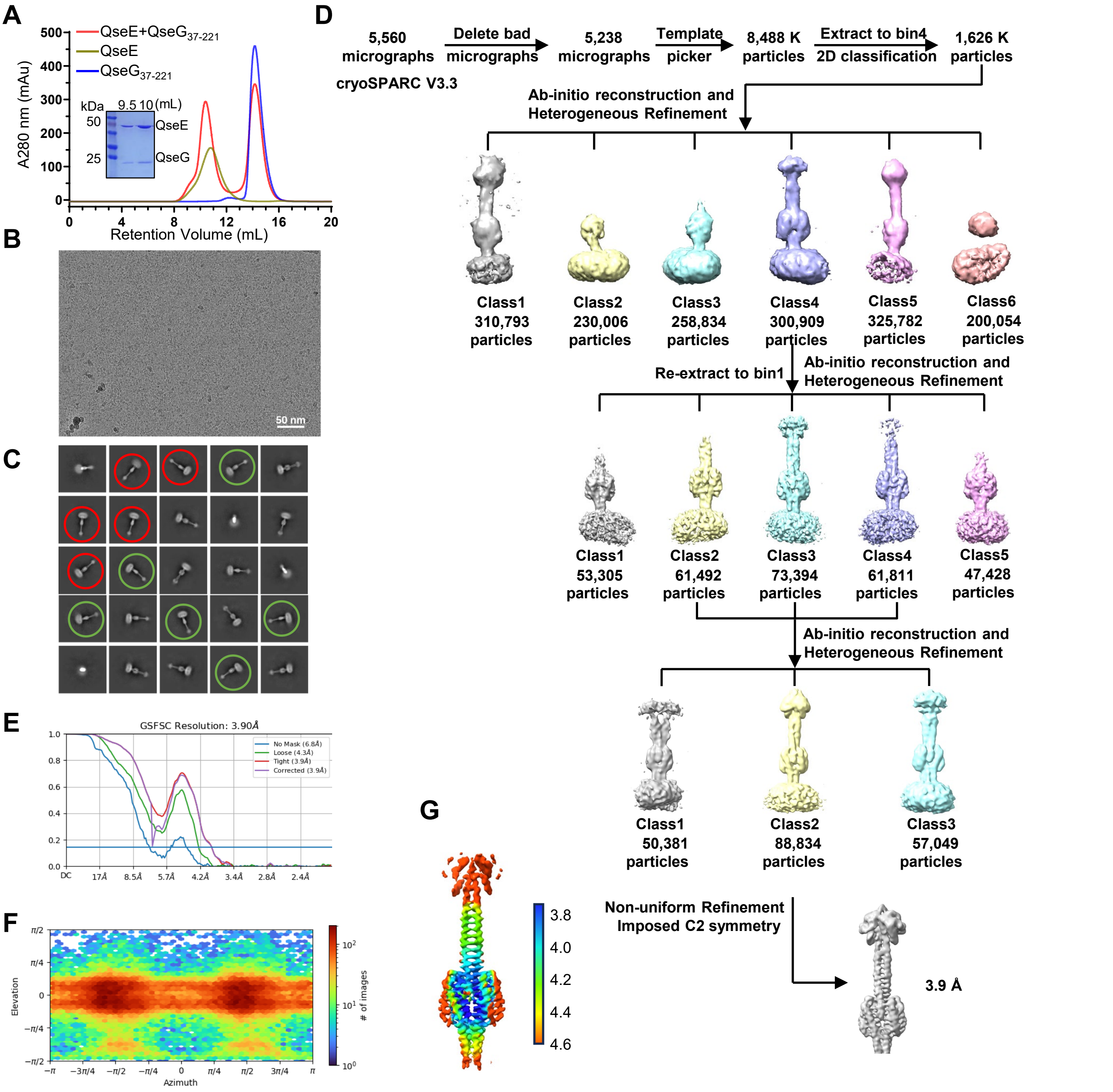

Supplemental Figure S3. Structure determination of $QseG_{37\text{-}221}$-$QseE_{FL}$ complex by cryo-EM.

(**A**) $QseG_{37\text{-}221}$-$QseE_{FL}$ complex for cryo-EM sample preparation was obtained by Size-exclusion chromatography analyses.

(**B**) A representative micrograph of QseG-QseE cryo-grids. The scale bar is 500 Å.

(**C**) Classification of QseG-QseE complex diversity in 2D: the red circles represent the classification where the QseG subunit is nearly vertical, while the green circles represent the classification where the QseG subunit is tilted at a certain angle.

(**D**) Schematic diagram of cryoEM data processing procedures for the QseG-QseE complex.

(**E**) Fourier Shell Coefficient (FSC) curve for 3D reconstruction maps of QseG-QseE complex.

(**F**) Angular distribution of QseG-QseE complex particle set.

(**G**) Local resolution of QseG-QseE complex displayed on the cryoEM map. The color code is shown in the color bar.

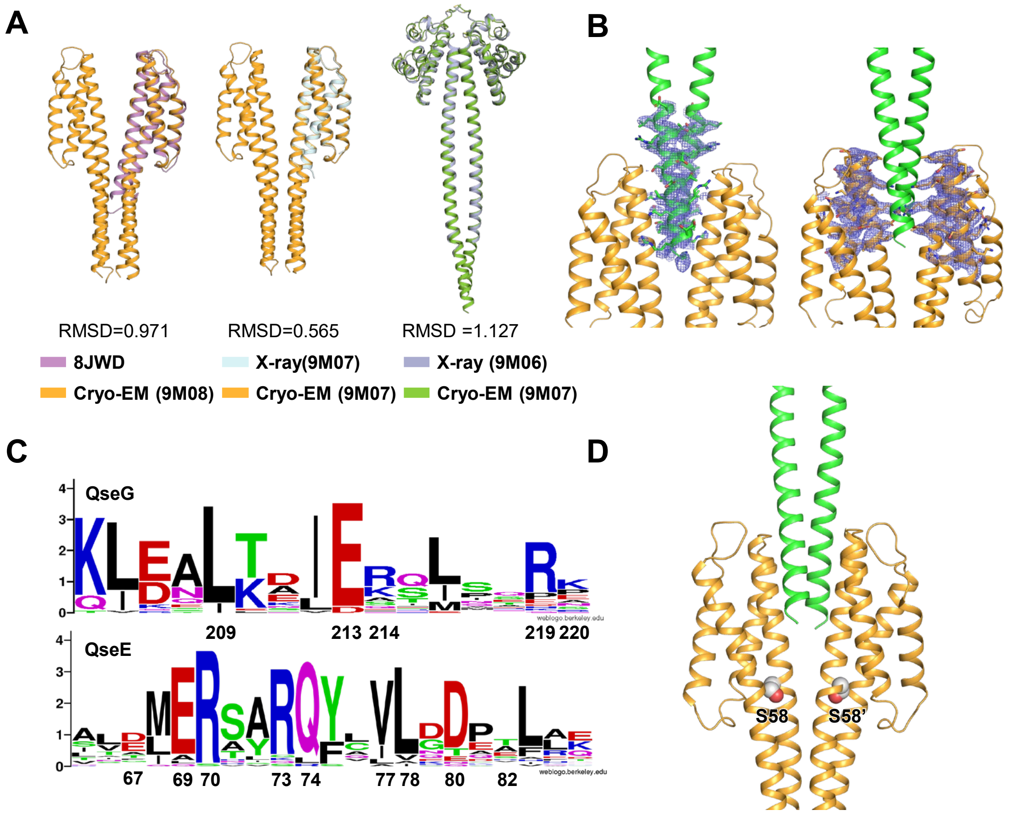

Supplemental Figure S4. Structural insights into the QseG–QseE interaction interface.

(**A**) Superposition of structures QseE and QseG from X-ray and Cryo-EM data.

(**B**) Electron microscopy density map of the QseE-QseG complex showing the interaction interface contours between the two proteins.

(**C**) Sequence logos are generated from alignments of the interface of QseG and QseE interaction across different bacterial families.

(**D**) S58 residues (shown as spheres) are located beneath the QseG-QseE interaction interface.

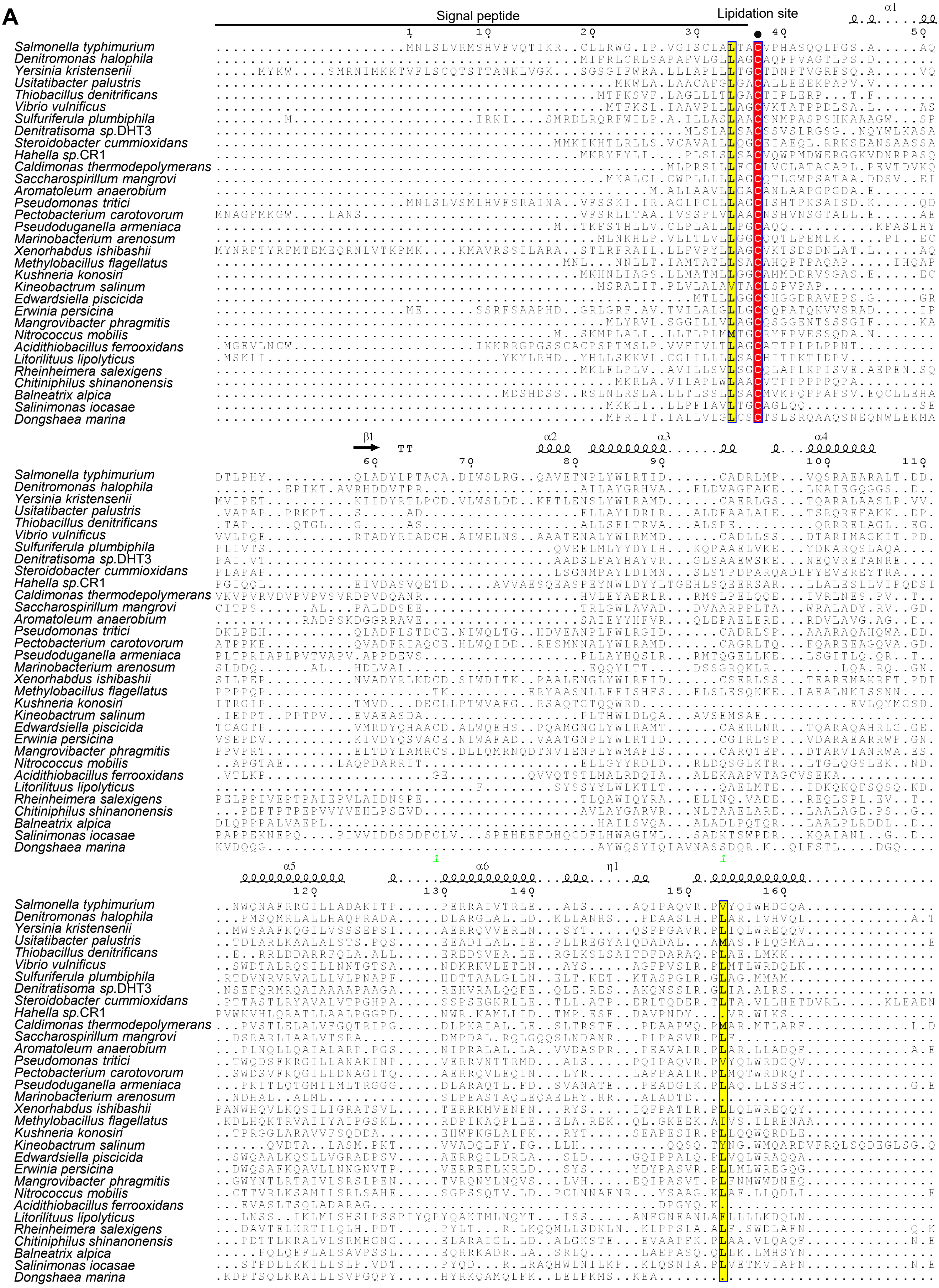
A
Signal peptide
Lipidation site
α1
β1
TT
α2
α3
α4
α5
α6
η1
Salmonella typhimurium
Denitromonas halophila
Yersinia kristensenii
Usitatibacter palustris
Thiobacillus denitrificans
Vibrio vulnificus
Sulfuriferula plumbiphila
Denitratisoma sp.DHT3
Steroidobacter cummioxidans
Hahella sp.CR1
Caldimonas thermodepolymerans
Saccharospirillum mangrovi
Aromatoleum anaerobium
Pseudomonas tritici
Pectobacterium carotovorum
Pseudoduganella armeniaca
Marinobacterium arenosum
Xenorhabdus ishibashii
Methylobacillus flagellatus
Kushneria konosiri
Kineobactrum salinum
Edwardsiella piscicida
Erwinia persicina
Mangrovibacter phragmitis
Nitrococcus mobilis
Acidithiobacillus ferrooxidans
Litorilituus lipolyticus
Rheinheimera salexigens
Chitiniphilus shinanonensis
Balneatrix alpica
Salinimonas iocasae
Dongshaea marina

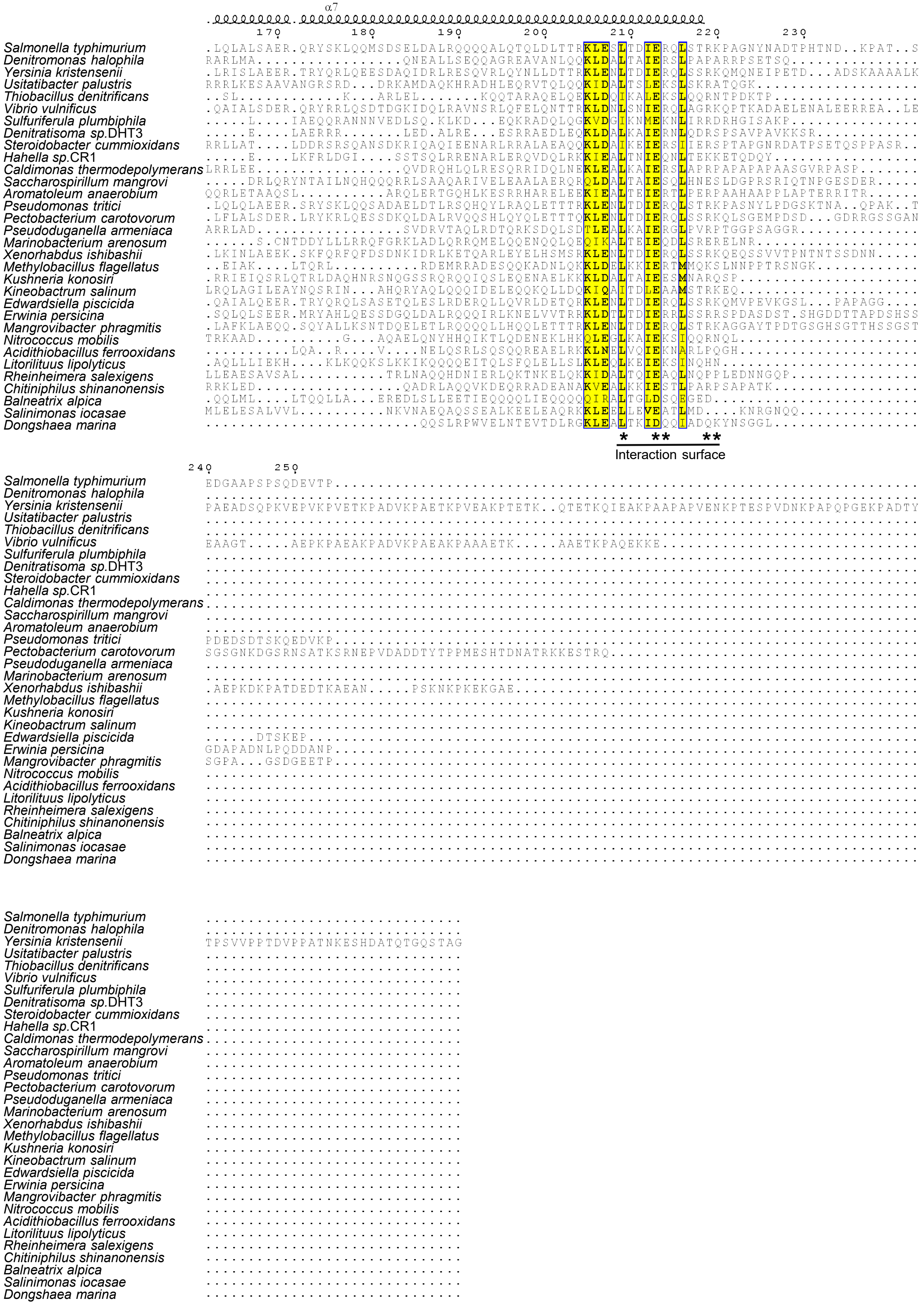

α7
170
180
190
200
210
220
230
240
250
Salmonella typhimurium
Denitromonas halophila
Yersinia kristensenii
Usitatibacter palustris
Thiobacillus denitrificans
Vibrio vulnificus
Sulfuriferula plumbiphila
Denitratisoma sp.DHT3
Steroidobacter cummioxidans
Hahella sp.CR1
Caldimonas thermodepolymerans
Saccharospirillum mangrovi
Aromatoleum anaerobium
Pseudomonas tritici
Pectobacterium carotovorum
Pseudoduganella armeniaca
Marinobacterium arenosum
Xenorhabdus ishibashii
Methylobacillus flagellatus
Kushneria konosiri
Kineobactrum salinum
Edwardsiella piscicida
Erwinia persicina
Mangrovibacter phragmitis
Nitrococcus mobilis
Acidithiobacillus ferrooxidans
Litorilituus lipolyticus
Rheinheimera salexigens
Chitiniphilus shinanonensis
Balneatrix alpica
Salinimonas iocasae
Dongshaea marina
Interaction surface

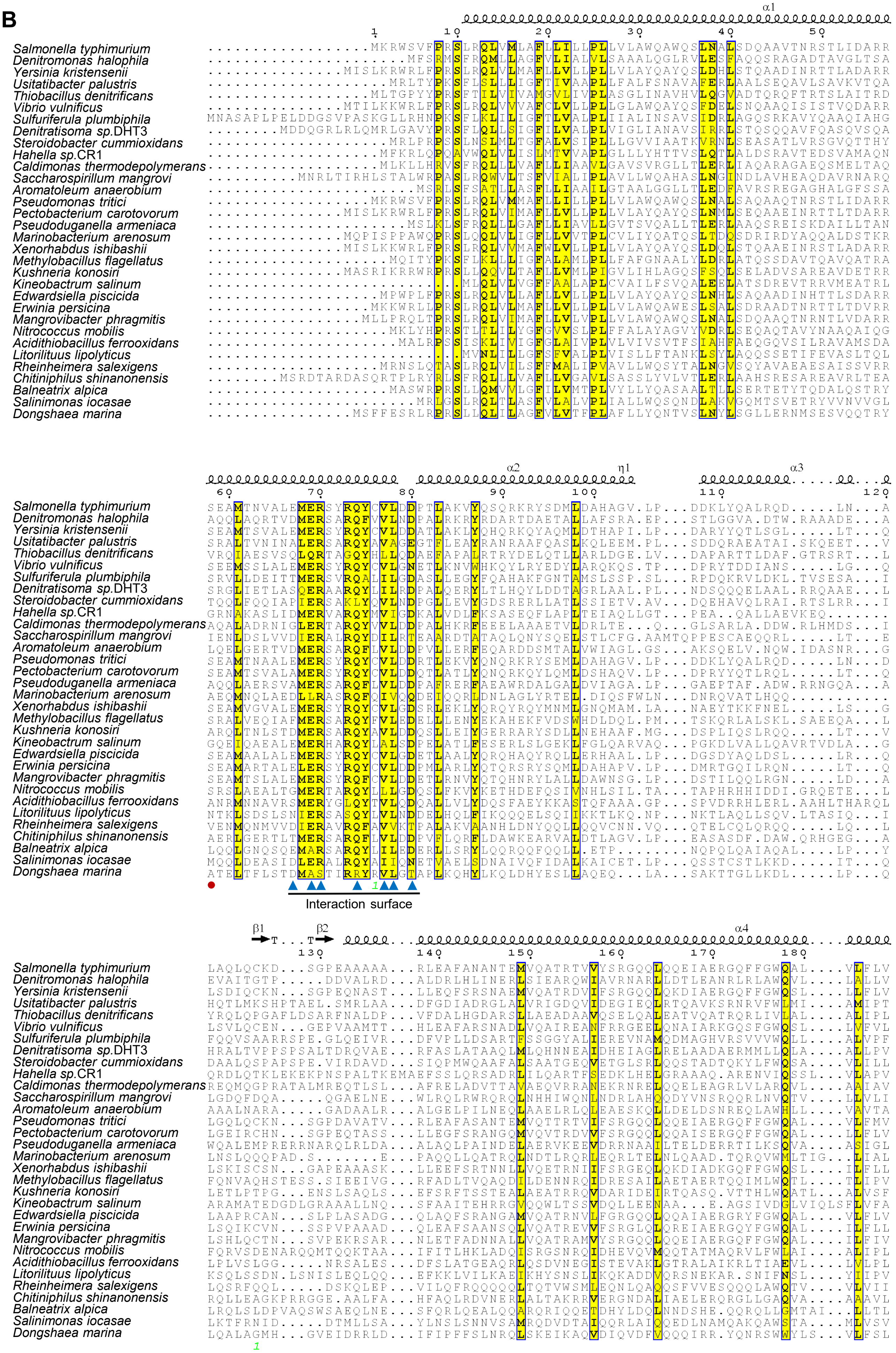
B
α1
α2
η1
α3
β1
β2
α4
Salmonella typhimurium
Denitromonas halophila
Yersinia kristensenii
Usitatibacter palustris
Thiobacillus denitrificans
Vibrio vulnificus
Sulfuriferula plumbiphila
Denitratisoma sp.DHT3
Steroidobacter cummioxidans
Hahella sp.CR1
Caldimonas thermodepolymerans
Saccharospirillum mangrovi
Aromatoleum anaerobium
Pseudomonas tritici
Pectobacterium carotovorum
Pseudoduganella armeniaca
Marinobacterium arenosum
Xenorhabdus ishibashii
Methylobacillus flagellatus
Kushneria konosiri
Kineobactrum salinum
Edwardsiella piscicida
Erwinia persicina
Mangrovibacter phragmitis
Nitrococcus mobilis
Acidithiobacillus ferrooxidans
Litorilituus lipolyticus
Rheinheimera salexigens
Chitiniphilus shinanonensis
Balneatrix alpica
Salinimonas iocasae
Dongshaea marina
Interaction surface

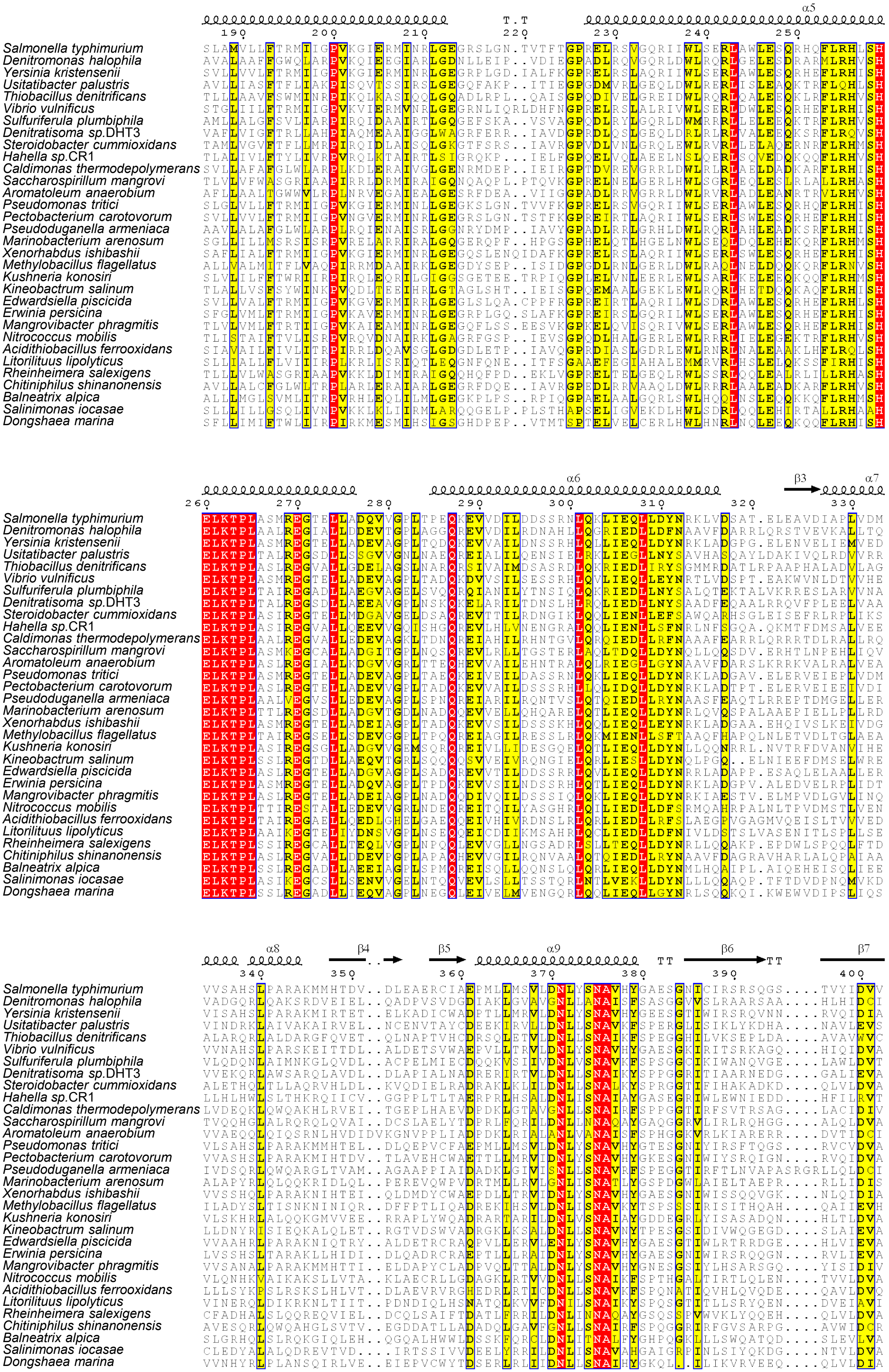
α5
190 200 210 220 230 240 250
T.T
Salmonella typhimurium
Denitromonas halophila
Yersinia kristensenii
Usitatibacter palustris
Thiobacillus denitrificans
Vibrio vulnificus
Sulfuriferula plumbiphila
Denitratisoma sp.DHT3
Steroidobacter cummioxidans
Hahella sp.CR1
Caldimonas thermodepolymerans
Saccharospirillum mangrovi
Aromatoleum anaerobium
Pseudomonas tritici
Pectobacterium carotovorum
Pseudoduganella armeniaca
Marinobacterium arenosum
Xenorhabdus ishibashii
Methylobacillus flagellatus
Kushneria konosiri
Kineobactrum salinum
Edwardsiella piscicida
Erwinia persicina
Mangrovibacter phragmitis
Nitrococcus mobilis
Acidithiobacillus ferrooxidans
Litorilituus lipolyticus
Rheinheimera salexigens
Chitiniphilus shinanonensis
Balneatrix alpica
Salinimonas iocasae
Dongshaea marina
α6 β3 α7
260 270 280 290 300 310 320 330
α8 β4 β5 α9 β6 β7
TT TT
340 350 360 370 380 390 400

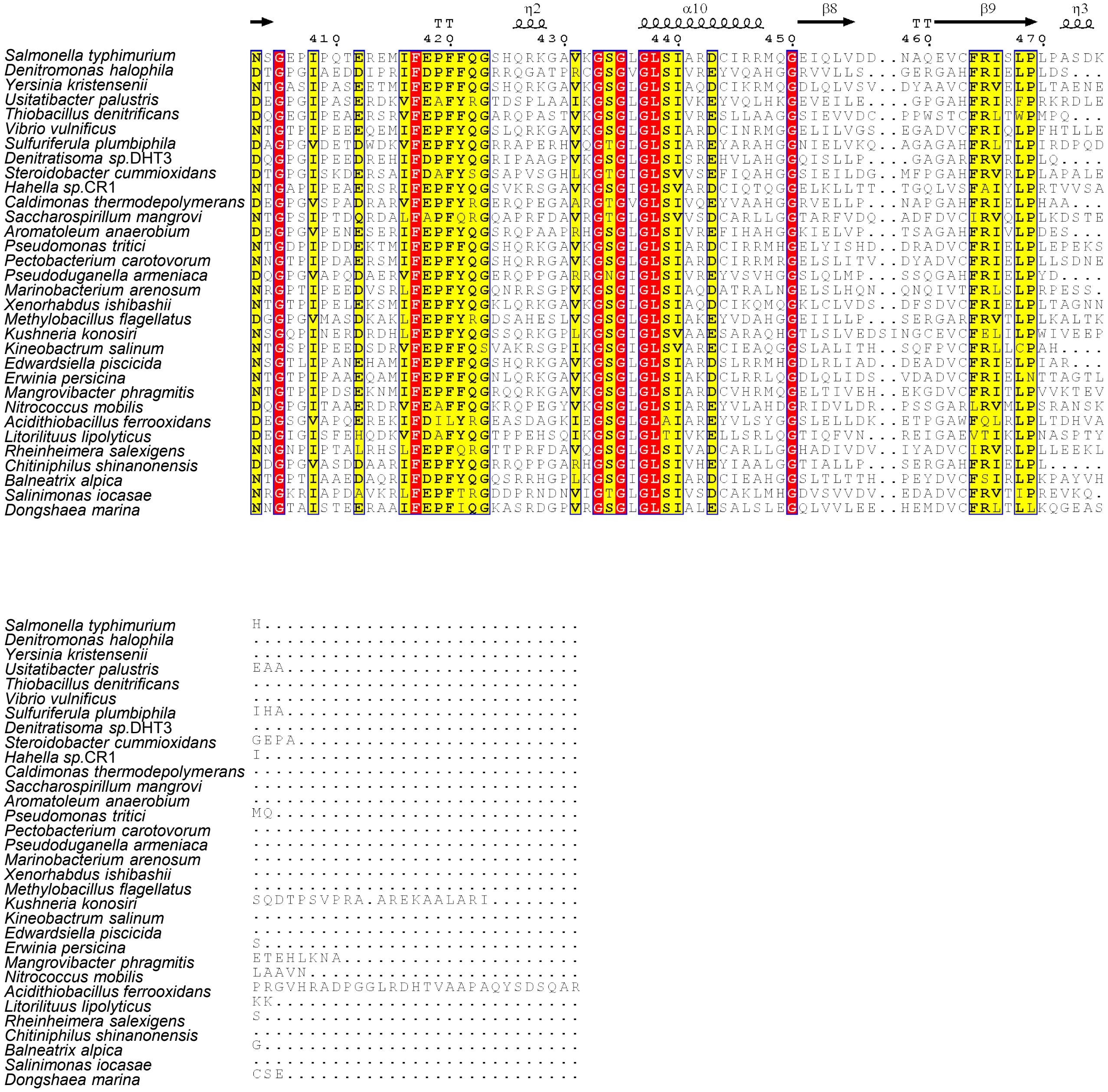

Supplemental Figure S5. Multiple sequence alignment of QseG and QseE reveals conserved residues.

(**A**-**B**) Multiple sequence alignment of QseG and QseE homologs across diverse bacterial families was performed using Clustal Omega and visualized with ESPript 3.0. Secondary structural elements of QseG and QseE are indicated above the sequences, arrows and spirals indicate β-strands and α-helices, respectively. Red and yellow backgrounds indicate identical and similar residue. The black circle marks the conserved esterification site C37 in QseG, while black asterisks denote key residues involved in QseG-QseE interaction below the sequences. The blue triangle symbols respectively denote amino acid residues in QseE involved in interactions at the QseG-QseE interface.

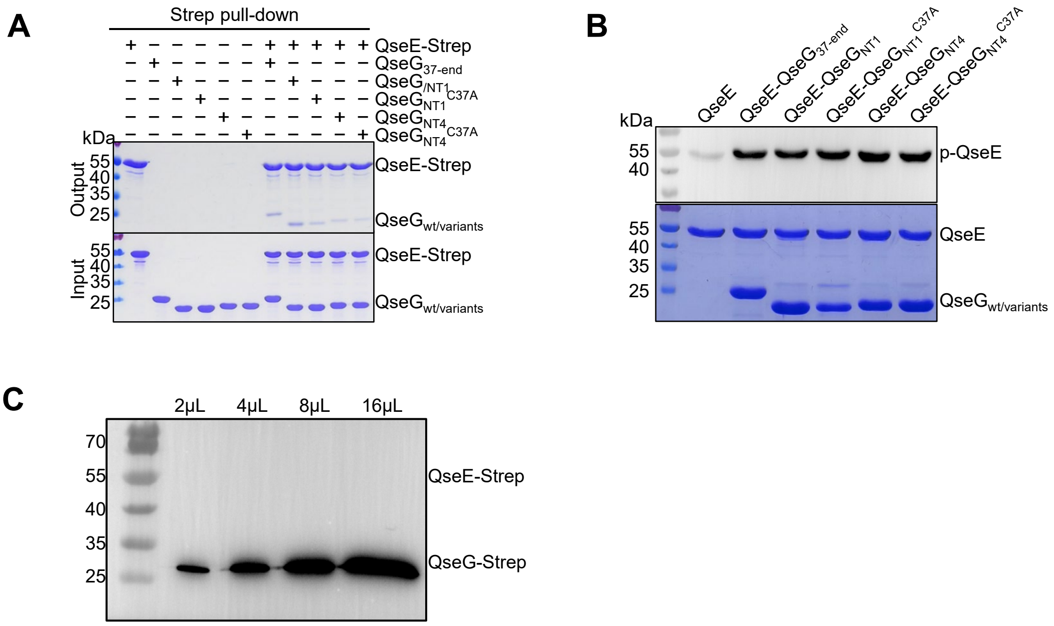

Supplemental Figure S6. Pull-down analysis and autokinase assay of QseG N-terminal mutations and the western blot characterization of the relative expression level of QseG and QseE in *Salmonella* typhimurium SL1344

(**A**) Pull-down analysis of strep-tagged QseE with QseG truncations and mutants (NT1, $NT1^{C37A}$, NT4, $NT4^{C37A}$).

(**B**) Autokinase assay of QseE induced by wide-type QseG and its N-terminal mutations. Upper panel shows the phosphorylation level of QseE (p-QseE), while lower panel displays the protein loading controls for both QseG and QseE.

(**C**) Western blot analysis of the relative expression levels of QseG and QseE. Identical Strep-tags were introduced *in situ* to both QseG and QseE in *Salmonella*, and their relative expression levels were evaluated by varying the sample loading amounts.

Supplemental Table S1. Bacterial strains used in this study

Strain Number	Relevant genotype/ description	Reference
S001	*E*. coli C43(DE3) for protein expression and purification	Lab collection
S002	cc118λ pir for plasmid amplification (*pir*-dependent)	Lab collection
S003	β2163Δnic35 DAP auxotrophic strain for conjugation	Lab collection
S004	*S*. typhimurium SL1344 WT	Lab collection
S005	*S*. typhimurium SL1344 Δ*qseG*	This paper
S006	*S*. typhimurium SL1344 QseG-strep	This paper
S007	*S*. typhimurium SL1344 QseG-strep+QseE-strep	This paper
S008	*S*. typhimurium SL1344 QseG NT4-strep	This paper
S009	*S*. typhimurium SL1344 Δ*qseG* :: P028 + P014	This paper
S010	*S*. typhimurium SL1344 Δ*qseG* :: P028 + P015	This paper
S011	*S*. typhimurium SL1344 Δ*qseG* :: P028 + P016	This paper
S012	*S*. typhimurium SL1344 Δ*qseG* :: P028 + P017	This paper
S013	*S*. typhimurium SL1344 Δ*qseG* :: P028 + P018	This paper
S014	*S*. typhimurium SL1344 Δ*qseG* :: P028 + P019	This paper
S015	*S*. typhimurium SL1344 Δ*qseG* :: P028 + P020	This paper
S016	*S*. typhimurium SL1344 Δ*qseG* :: P028 + P021	This paper
S017	*S*. typhimurium SL1344 Δ*qseG* :: P028 + P022	This paper
S018	*S*. typhimurium SL1344 Δ*qseG* :: P028 + P023	This paper
S019	*S*. typhimurium SL1344 Δ*qseG* :: P028 + P024	This paper
S020	*S*. typhimurium SL1344 Δ*qseG* :: P028 + P025	This paper
S021	*S*. typhimurium SL1344 Δ*qseG* :: P028 + P026	This paper
S022	*S*. typhimurium SL1344 QseG-strep :: P027	This paper
S023	*S*. typhimurium SL1344 QseG NT4-strep :: P027	This paper
S024	*S*. typhimurium SL1344 Δ*qseG* :: P027	This paper

Supplemental Table S2. Plasmid used in this study

Plasmid Number	Plasmid	Reference
P001	pET22b-pelB-Strep-TEV-QseG$_{37-181}$	This paper
P002	pET22b-pelB-Strep-TEV-QseG$_{37-193}$	This paper
P003	pET22b-pelB-Strep-TEV-QseG$_{37-214}$	This paper
P004	pET22b-pelB-Strep-TEV-QseG$_{37-221}$	This paper
P005	pET22b-pelB-Strep-TEV-QseG$_{37\text{-}end}$	This paper
P006	pET22b-pelB-Strep-TEV-QseG$_{37\text{-}end}$ ^{L209D}	This paper
P007	pET22b-pelB-Strep-TEV-QseG$_{37\text{-}end}$ ^{E213A}	This paper
P008	pET22b-pelB-Strep-TEV-QseG$_{37\text{-}end}$ ^{R214A}	This paper
P009	pET22b-pelB-Strep-TEV-QseG$_{37\text{-}end}$ ^{R219A}	This paper
P010	pET22b-pelB-Strep-TEV-QseG$_{37\text{-}end}$ ^{K220A}	This paper
P011	pET22b-pelB-Strep-TEV-QseG$_{NT1}$	This paper
P012	pET22b-pelB-Strep-TEV-QseG$_{NT1}^{C37A}$	This paper
P013	pET22b-pelB-Strep-TEV-QseG$_{NT4}$	This paper
P014	pET22b-pelB-Strep-TEV-QseG$_{NT4}$ ^{C37A}	This paper
P015	pET28b-QseE-Strep	This paper
P016	pET28b-His-sumo-QseE$_{1-196}$-Strep	This paper
P017	pET28b-His-sumo-QseE$_{38-171}$-Strep	This paper
P018	pBAD-vector	This paper
P019	pBAD-QseG-strep	This paper
P020	pBAD-QseG$_{1-181}$-strep	This paper
P021	pBAD-QseG^{C37A}-strep	This paper
P022	pBAD-QseG^{L209D}-strep	This paper
P023	pBAD-QseG^{E213A}-strep	This paper
P024	pBAD-QseG^{R214A}-strep	This paper
P025	pBAD-QseG^{R219A}-strep	This paper
P026	pBAD-QseG^{K220A}-strep	This paper
P027	pBAD-QseG$_{NT1}$-strep	This paper
P028	pBAD-QseG$_{NT1}^{C37A}$-strep	This paper
P029	pBAD-QseG$_{NT4}$-strep	This paper
P030	pBAD-QseG$_{NT4}^{C37A}$-strep	This paper
P031	pBAD-QseE^{D80X}-HA	This paper
P032	pSC101-P$_{glmY}$-EGFP	This paper
P033	pSB890-ΔqseG	This paper
P034	pSB890-*qseG*-strep	This paper
P035	pSB890-*qseG* NT4-strep	This paper

Supplemental Table S3. List of oligonucleotides used in this study

ID	Sequence (5' to 3')	Purpose
P001-F	GCAAACTCCAGCAAATGTCATAAGATCCGGCTGCTAAC	P001
P001-R	TGACATTTGCTGGAGTTTGCTATAACGCTGACGTTC	P001
P002-F	CGCTGCGCCAGCAGCAACAGTAAGATCCGGCTGCTAAC	P002
P002-R	CTGTTGCTGCTGGCGCAGCGCATCCAGCTCACTATCT	P002
P003-F	GTTTAACCGATATTGAGCGCTAAGATCCGGCTGCTAAC	P003
P003-R	GCGCTCAATATCGGTTAAACTCTCCAGCTTACGGGTG	P003
P004-F	GATCCGGCTGCTAACAAAGC	P004
P004-R	CTTTGTTAGCAGCCGGATCTTACGGTTTTCGTGTCGAAAG	P004
P005-VF	GATCCGGCTGCTAACAAAGC	P005
P005-VR	TCCCTGAAAATACAGGTTTTCT	P005
P005-F	TTTGTTAGCAGCCGGATCTTATGGCGTTACCTCATCTTG	P005
P005-R	TCGAGAAAGAAAACCTGTATTTTCAGGGATGTGTTCCCCATGCTTCAC	P005
P006-F	CACCCGTAAGCTGGAGAGTGACACCGATATTGAG	P006/P022
P006-R	GTCACTCTCCAGCTTACGGGTGGTAAG	P006/P022
P007-F	TTTAACCGATATTGCGCGCCAGCTTTCG	P007/P023
P007-R	CGCAATATCGGTTAAACTCTCCAG	P007/P023
P008-F	GTTTAACCGATATTGAGGCCCAGCTTTCGAC	P008/P024
P008-R	GGCCTCAATATCGGTTAAACTCTCCAGCT	P008/P024
P009-F	TGAGCGCCAGCTTTCGACAGCAAAACCGGCAGGGAAT	P009/P025
P009-R	TGCTGTCGAAAGCTGGCGCTCAATATCGGTTAAAC	P009/P025
P010-F	GCCAGCTTTCGACACGAGCACCGGCAGGGAATTAC	P010/P026
P010-R	TGCTCGTGTCGAAAGCTGGCGCTCAATATCGGT	P010/P026
P011-F	GTATTTTCAGGGATGTGTTCCCCATTATCAACTGGCG	P011
P011-R	AACACATCCCTGAAAATACAGG	P011
P014-F	CAGCAACTGCCCGGTAGCGCGGATTATCTCCCGACG	P014
P014-R	GCTACCGGGCAGTTGCTGTGAAGCATGGGGAAC	P014
P015-VF	TGGAGCCACCCGCAGTTCGAGAAATGAGATCCGGCTGCTAAC	P015
P015-VR	CATGGTATATCTCCTTCT	P015
P015-F	AGAAGGAGATATACCATGAAGCGCTGGTCTGTTTTC	P015
P015-R	GAACTGCGGGTGGCTCCACGAACCGTGTTTGTCAGATGCAGG	P015
P016-VF	CACTGAGATCCGGCTGCTAACAAAG	P016
P016-VR	GGATCCACCAATCTGTTCTCTGTGAGCCTC	P016
P016-F	GAACAGATTGGTGGATCCATGAAGCGCTGGTCTGTTTTC	P016
P016-R	AGCAGCCGGATCTCAGTGTTACATGCGGGTAAAAAGTAG	P016
P017-F	GAACAGATTGGTGGATCCAACGCGTTAAGCGATCAG	P017
P017-R	AGCAGCCGGATCTCAGTGTTAGCGCTCGGCGATTTCTTG	P017
P019-VF	TGGAGCCACCCGCAGTTC	P019

P019-VR	GGTGAATTCCTCCTGCTAGC	P019
P019-F	TAGCAGGAGGAATTCACCATGAATTTAAGCCTGGTG	P019
P019-R	GAACTGCGGGTGGCTCCATGGCGTTACCTCATCTTGT	P019
P020-F	AAACTCCAGCAAATGTCATGGAGCCACCCGCAGTTCG	P020
P020-R	TGACATTTGCTGGAGTTTGCTATAACGCTGACGT	P020
P021-F	CGTGTCTGGCGCTGACCGCAGCTGTTCCCCATGCTTCAC	P021/P030
P021-R	AGCTGCGGTCAGCGCCAGACACGATATGCCCACCGGT	P021/P030
P028-F	TGTCTGGCGCTGACCGCAGCTGTTCCCCATTATCAACTG	P028
P028-R	AGCTGCGGTCAGCGCCAGACACGATATGCCCACCGGT	P028
P030-F1	TAGCAGGAGGAATTCACCATGAAGCGCTGGTCTGT	P030
P030-R1	GACTCTAGAGAGATTTAAGCGTAGTCTGGGACGTCGTATG GGTAGTGTTTGTCAGATGC	P030
P030-F2	GTATTGCGTGCTTGACTAGCCGACGCTGGCAAAAG	P030
P030-R2	CTAGTCAAGCACGCAATACTGTCGGTAGCTTCG	P030
P032-VF	TAAAAGCTTCGAGGTGAAGAC	P032
P032-VR	ATGTATATCTCCTTCTTAAATC	P032
P032-F1	TAAGAAGGAGATATACATTTAGCCCGATAGCCATTAAG	P032
P032-R1	CTCCTTGGTTACCATTATGCAGT	P032
P032-F2	CTGCATAATGGTAACCAAGGAGATATACATATGAGCAAAGG TGAAGAAC	P032
P032-R2	TTCACCTCGAAGCTTTTACTTATACAGTTCATCCAT	P032
P033-VF	GGATCCCCCGGGCTGCAGTTCACT	P033/P034/ P035
P033-VR	CCGCGGTGGGGCTTTTTATTGAGC	P033/P034/ P035
P033-F1	CAATAAAAAGCCCCACCGCGGAAATGGTGCAGGCGACGC	P033
P033-R1	GGCGTTACCTCATTAGTGTTTGTCAGATGCAGGCAATGGC	P033
P033-F2	CAAACACTAATGAGGTAACGCCATGATAAGCCG	P033
P033-R2	CTGCAGCCCGGGGGATCCGCTGTGCCGACTGTCTGAGC	P033
P034-F1	CAATAAAAAGCCCCACCGCGGCCGAAAGAGAAATGATTTT TGAG	P034
P034-R1	CGAACTGCGGGTGGCTCCATGGCGTTACCTCATCTTGTGA CG	P034
P034-F2	GGAGCCACCCGCAGTTCGAAAAGTAAGAGGTAACGCCAT GATAAG	P034
P034-R2	CTGCAGCCCGGGGGATCCCGAACAAACGGTTTATGCCGC	P034
P035-F1	CAATAAAAAGCCCCACCGCGGGGTGCTATTTCTGGTGAGC	P035
P035-R1	GGGAGATAATCCGCGCTACCGGGCAGTTGCTGTGAAG	P035
P035-F2	TAGCGCGGATTATCTCCCGACGGCGTGCGCGGATATCTG	P035
P035-R2	CTGCAGCCCGGGGGATCCCAGCGCTTCATCTATCGC	P035

Supplemental Table S4. Cryo-EM data collection, refinement and validation statistics

	QseG-QseE complex
**Data collection**	
EM equipmen	Titan Krios (Thermo Fisher Scientific)
Voltage (kV)	300
Electron exposure (e-/Å2)	50
Defocus range (μm)	-0.5 ~ -2.0
Pixel size (Å)	1.06
Number of movies	5560
**Reconstruction**	
Software	cryoSPARC
Symmetry imposed	C2
Initial particle images (no.)	8,488,343
Final particles images (no.)	88,834
Map resolution (Å)	3.9
Map sharpening B factor	125.2
FSC threshold	0.143
Local map resolution range (Å)	3.8-4.6
**refinement**	
Software	PHENIX & COOT
Model resolution (Å)	3.9
Model composition	
Non-hydrogen atoms	5085
Protein residues	638
R.m.s deviations	
Bond length (Å)	0.003
Bond angles (°)	0.754
Ramachandran plot	
Favored (%)	98.41
Allowed (%)	1.59
Outliers (%)	0
**Validation**	
MolProbity score	1.7
Clashscore	15.58
Poor rotamers (%)	0.38
PDB code	9M08
EMDB code	63539

Supplemental Table S5. QseG and QseE crystal data processing and structure refinement statistics

	$QseG_{37-221}$	$QseE_{38-171}$
PDB ID	9M06	9M07
**Data collection**		
Wavelength	0.9785	0.9785
Resolution range	50.00 – 2.10 (2.18 – 2.10)	50.00 – 2.40 (2.49 – 2.40)
Space group	C1 2 1	P41212
a b c (Å)	90.09 41.00 126.66	55.45 55.45 88.12
α β γ (°)	90.00 103.18 90.00	90.00 90.00 90.00
Unique reflections	26629 (2506)	5687(541)
Completeness (%)	99.1 (92.9)	99.9(99.8)
Mean I/sigma(I)	13.5 (2.2)	30(6)[§]
Wilson B-factor	25.1	33.1
R-meas	0.130(0.628)	0.119(0.522)
CC1/2	0.977 (0.754)	0.992(0.985)
Data redundancy	6.0 (4.1)	23.5(22.1)
**Refinement**		
Resolution range	33.27 - 2.10 (2.18 - 2.10)	24.8 - 2.40 (2.486 - 2.4)
Reflections used in refinement	25813 (2063)	5358 (342)
Reflections used for R-free	1994 (159)	536 (34)
R-work	0.2102 (0.2846)	0.2230 (0.2765)
R-free	0.2342 (0.2920)	0.2726 (0.3230)
Number of non-hydrogen atoms	2518	877
macromolecules	2306	846
ligands	23	0
solvent	189	31
Protein residues	286	108
RMS (bonds)	0.002	0.003
RMS (angles)	0.42	0.59
Ramachandran favored (%)	98.94	100
Ramachandran allowed (%)	1.06	0.00
Ramachandran outliers (%)	0.00	0.00
Rotamer outliers (%)	0.00	0.00
Clashscore	2.58	8.34
Average B-factor	31.05	39.98
macromolecules	30.76	39.97
ligands	40.41	40.15

*Values in parentheses are for highest-resolution shell.

[§]Due to ice contamination, the resolution of this dataset was limited to 2.4 Å, at which point the I/σ in the last resolution shell exceeded the conventional cutoff value of 2.
